# Sonic hedgehog medulloblastoma cells in co-culture with cerebellar organoids converge towards in vivo malignant cell states

**DOI:** 10.1093/noajnl/vdae218

**Published:** 2024-12-13

**Authors:** Max J van Essen, Alina Nicheperovich, Benjamin Schuster-Böckler, Esther B E Becker, John Jacob

**Affiliations:** Kavli Institute for Nanoscience Discovery, University of Oxford, Oxford, UK; Nuffield Department of Clinical Neurosciences, University of Oxford, Oxford, UK; Ludwig Institute for Cancer Research, Nuffield Department of Medicine, University of Oxford, Oxford, UK; Big Data Institute, Li Ka Shing Centre for Health Information and Discovery, University of Oxford, Oxford, UK; Ludwig Institute for Cancer Research, Nuffield Department of Medicine, University of Oxford, Oxford, UK; Kavli Institute for Nanoscience Discovery, University of Oxford, Oxford, UK; Nuffield Department of Clinical Neurosciences, University of Oxford, Oxford, UK; Nuffield Department of Clinical Neurosciences, University of Oxford, Oxford, UK

**Keywords:** single-cell transcriptomics, human induced pluripotent stem cells, human cerebellar organoids, medulloblastoma cell lines, sonic hedgehog medulloblastoma

## Abstract

**Background:**

In the malignant brain tumor sonic hedgehog medulloblastoma (SHH-MB) the properties of cancer cells are influenced by their microenvironment, but the nature of those effects and the phenotypic consequences for the tumor are poorly understood. The aim of this study was to identify the phenotypic properties of SHH-MB cells that were driven by the nonmalignant tumor microenvironment.

**Methods:**

Human induced pluripotent cells (iPSC) were differentiated to cerebellar organoids to simulate the nonmaliganant tumor microenvironment. Tumor spheroids were generated from 2 distinct, long-established SHH-MB cell lines which were co-cultured with cerebellar organoids. We profiled the cellular transcriptomes of malignant and nonmalignant cells by performing droplet-based single-cell RNA sequencing (scRNA-seq). The transcriptional profiles of tumor cells in co-culture were compared with those of malignant cell monocultures and with public SHH-MB datasets of patient tumors and patient-derived orthotopic xenograft (PDX) mouse models.

**Results:**

SHH-MB cell lines in organoid co-culture adopted patient tumor-associated phenotypes and showed increased heterogeneity compared to monocultures. Subpopulations of co-cultured SHH-MB cells activated a key marker of differentiating granule cells, *NEUROD1* that was not observed in tumor monocultures. Other subpopulations expressed transcriptional determinants consistent with a cancer stem cell-like state that resembled cell states identified in vivo.

**Conclusions:**

For SHH-MB cell lines in co-culture, there was a convergence of malignant cell states towards patterns of heterogeneity in patient tumors and PDX models implying these states were non-cell autonomously induced by the microenvironment. Therefore, we have generated an advanced, novel in vitro model of SHH-MB with potential translational applications.

**Key Points**

- SHH-MB cell lines in cerebellar organoid co-culture recapitulate certain in vivo malignant cell states.
- The organoid microenvironment non-cell autonomously regulates SHH-MB cell states.

**Importance of the Study**

Traditional in vitro models of SHH-MB have not performed well in translational studies that aimed to identify prognostic markers or targetable molecules. Novel in vitro models of SHH-MB that can be generated under defined conditions, that are better able to recapitulate patient tumors, and that are scalable would be invaluable in accelerating translational research. We co-cultured long-established SHH-MB lines with nonmalignant cerebellar organoids to simulate the tumor microenvironment. We show that malignant cells in co-culture resemble patient tumors and PDX models more closely than traditional in vitro models. These changes appear to be driven by the emergence of cellular states that are not observed in simpler in vitro models. Our data support the view that SHH-MB cellular states are strongly influenced by cell-extrinsic mechanisms originating in the nonmalignant compartment.

Medulloblastoma (MB), a heterogeneous cerebellar tumor consisting of 4 major subtypes is the commonest malignant brain tumor of childhood,^1^ of which nearly a third are classed as Sonic Hedgehog medulloblastoma (SHH-MB).^2^ During development, SHH acting as a mitogen promotes massive expansion of the upper rhombic lip-derived cerebellar granule cell population in the external granule layer.^3^ Somatic or germ-line mutations and focal somatic copy number alterations in SHH pathway tumor suppressors or oncogenes are the commonest events leading to aberrant SHH pathway activation and tumorigenesis in granule progenitors.^2^ High-risk variants of SHH-MB still carry a dismal prognosis, which emphasizes the importance of gaining a greater understanding of how tumors progress so that improved therapies can be developed.

In patients and mouse models, SHH-MB contains SOX2^+^ tumor-propagating neural stem-like cells, equivalent to cancer stem cells (CSC) and more differentiated cells.^2^ By hijacking mechanisms of self-renewal in neural stem cells (NSC) and pluripotent stem cells, CSC can replenish tumors in transplantation assays.^4^ During mammalian neurogenesis, initial ubiquitous SOX2 expression in the early embryonic central nervous system attenuates as the balance shifts from self-renewal to differentiation.^5^ By contrast, in SHH-MB persistent SOX2-expressing granule progenitor-derived CSC can reconstitute tumors after therapy and support a hierarchical model of tumorigenesis.^6^ Furthermore, cross-talk between the microenvironment and other brain tumor types suggests that cell-extrinsic factors could also influence malignant cell states.^4^ However, the complexity of the in vivo tumor microenvironment poses a challenge in discerning the involvement of distinct nonmalignant components.

To address how the microenvironment affects tumor progression, we sought to evaluate the phenotypes of SHH-MB cells using single-cell transcriptomics, under defined conditions that we anticipated might simulate in vivo tumor growth. Our objective was to determine whether, in SHH-MB, where malignant cell states have typically been considered cell-autonomous, our novel methodology could uncover microenvironmental dependencies that influence cell states. To this end, we leveraged cerebellar organoid differentiation from human induced pluripotent stem cells (iPSC) through their co-culture with 2 SHH-MB cell lines^7^ that were compared with conventional in vitro tumor monocultures. Organoids can be grown in xeno-free culture medium and they contain defined neuronal and glial cell types. Although lacking immune cells or blood vessels, they provide more physiological conditions than conventional in vitro models enabling the evaluation of tumor cell phenotypes in a simulated native microenvironment.^8^

## Materials and Methods

### Organoid Differentiation

Cerebellar organoid differentiation was performed as described previously.^9^ In brief, human iPSC line AH017-3 cells^10^ were detached by incubation with pre-warmed TrypLE for 5 minutes. Cells were collected by centrifugation and resuspended in induction medium (50% IMDM, 50% F12, 7 ug/mL Insulin, 5 mg/mL BSA, 1X Chemically defined Lipid concentrate, 450 uM Monothioglycerol, 15 ug/mL Apo-transferrin, 1X Penicillin/Streptomycin) at a final concentration of 100 000 cells/ml. 10 000 cells per well were plated in an ultra-low-attachment V-bottom 96-well plate and returned to the incubator. After 2 days, FGF2 was added at a final concentration of 50 ng/ml. One-third medium change was performed on day 7 and full medium change on day 14. On this day, the organoids were also transferred into a low-attachment 48-well plate. On day 21, the medium was changed to a differentiation medium (Neurobasal + 1% N2 supplement + 1% GlutaMAX + 1X Penicillin/Streptomycin), and subsequent full medium changes were performed on days 28 and 35. After day 35, the medium was changed twice per week.

### Medulloblastoma Cell Lines, Tumor Spheroids, and Organoid-Tumor Spheroid Co-culture

**Figure 1.**
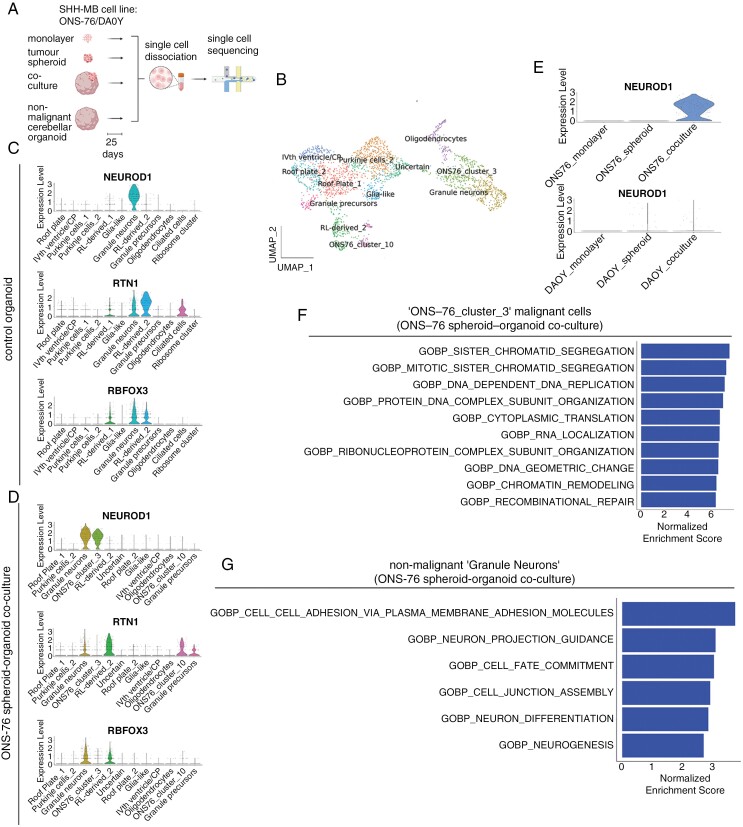
Upregulation of *NEUROD1* in ONS-76 cells in co-culture compared to monolayer or tumor spheroid growth conditions. (A) Schematic of experimental workflow. This figure was created with BioRender.com. (B) UMAP visualization of single-cell transcriptomes of ONS-76 cells in co-culture identified 2 malignant cell clusters annotated as ‘ONS76_cluster_3’ and ‘ONS76_cluster_10’. (C) Violin plot showing co-expression of *NEUROD1*, *RTN1,* and *RBFOX3* in the ‘Granule neurons’ cluster of the control organoid. (D) Violin plot of expression of *NEUROD1,* and absent *RTN1* and *RBFOX3* expression in ‘ONS76_cluster_3’ in the ONS-76 tumor spheroid-organoid co-culture. (E) Violin plots of the expression of *NEUROD1* in ONS-76 cells (upper panel) and DAOY cells (lower panel) grown as monolayers, tumor spheroids or in tumor spheroid-organoid co-culture. (F) fgsea gene set enrichment of upregulated gene ontology (GO) biological pathway terms evaluated by normalized enrichment scores of the *NEUROD1*^+^ cluster of malignant cells (‘ONS76_cluster_3’) in the ONS-76 tumor spheroid-organoid co-culture sample. (G) fgsea gene set enrichment of upregulated GO biological pathway terms evaluated by normalized enrichment scores of nonmalignant *NEUROD1*^*+*^ cells (‘Granule neurons’) in the ONS-76 tumor spheroid-organoid co-culture sample.

DAOY-GFP cells (gift from Vincenzo D’Angiolella, DAOY cell line originally purchased from ATCC) were grown in monolayer culture in MEM, 1% GlutaMAX, 1X Penicillin/Streptomycin and 1% FBS (cell line authentication in Supplementary Table 1). ONS-76-Luciferase-GFP cells (gift from Louis Chesler; ONS-76 cell line originally purchased from JCRB Cell Bank by Steven Clifford) were grown in monolayer culture in RPMI + 1% GlutaMAX + 1X Penicillin/Streptomycin + 1% FBS (Supplementary Table 1). The medium was changed twice per week. To generate tumor spheroids, cells were detached by incubation with pre-warmed TrypLE for 5 minutes and collected by centrifugation. Cells were resuspended in differentiation medium at a concentration of 200 000 cells/ml and plated in an ultra-low-attachment V-bottom 96-well plate at 20 000 cells per well. In preparation for cerebellar organoid-tumor spheroid co-culture, 1 week prior to the start of co-culture, tumor spheroids were generated as described above. One tumor spheroid was placed with 1 day 35 organoid in a low-attachment 24-well plate in an identical differentiation medium. The size ratio of the organoid to the tumor spheroid of approximately 5-to-one ensured that the organoid microenvironment could encompass the tumor spheroid to facilitate their interaction. To allow the fusion of the tumor spheroid and the organoid, the plate was incubated for 2 days at a 45-degree angle. The medium was changed twice per week and the cells were maintained in co-culture, alongside tumor spheroid and tumor monolayer cultures for a further 25 days (Figure 1A).

### Single Cell Isolation

Day 60 organoids and organoid-tumor co-culture samples were collected using a wide bore pipette tip and placed in a 6-well plate. Five tumor spheroid-organoid co-cultures for each cell line and a similar number of control organoids were each cut into 4 pieces and placed in an Eppendorf tube. After the removal of the excess medium, 200 μl of pre-warmed (to 37°C) Neuron isolation enzyme was added and the tube was placed in a 37°C water bath. Organoids were incubated with the enzyme for 30 minutes with intermittent agitation. The enzyme was removed and organoids were washed once with HHGN medium (HBSS + 2.5mM HEPES + 35mM Glucose + 4mM NaHCO_3_). Organoids were then dissociated in the differentiation medium by gentle trituration. Afterward, the cell suspension was enriched for viable cells using the dead cell removal kit (Miltenyi Biotec) following the manufacturers' protocol. Cell viability was determined using Trypan Blue staining and counted using an automated cell counter (Applied Biosystems Countess 3). Cells were centrifuged at 300 × *g* for 5 minutes and resuspended in the appropriate volume of resuspension buffer (HBSS + 0.04% BSA) to a final concentration of 2000 cells/μl before proceeding with single-cell RNA sequencing. Thirty tumor spheroids were collected using a wide-bore pipette tip and placed in an Eppendorf tube. Excess medium was removed and 200 μl of pre-warmed Neuron isolation enzyme (Gibco) was added. The tube was placed in a 37°C water bath for 20 minutes and agitated every 5 minutes. Subsequent processing was performed following the same steps as described above. DAOY and ONS-76 cell monolayer cultures were dissociated by incubation with pre-warmed TrypLE for 5 minutes. TrypLE was then diluted in PBS before centrifugation at 400 × *g* for 5 minutes to collect the cells. Cells were washed once in the differentiation medium before processing as described above. Single-cell barcoding and reverse transcription of poly-A mRNA was performed using the 10X Chromium controller (3’ version 3.1 chemistry) and sequencing of single-cell libraries was performed on an Illumina NovaSeq6000 sequencing system according to the manufacturer’s instructions.

### Data Pre-processing and Analysis

Initial sequencing data exploration was performed using Cell Ranger v6.1.1. For each dataset, we removed cells with 10% or higher mitochondrial gene expression^11^ and genes expressed in fewer than 10 cells. Sequencing confirmed the greater proportion of nonmalignant to malignant cell types in co-culture which was expected given the relative sizes of the organoid and tumor spheroid (Supplementary Table 2). Downstream analysis largely implemented the workflow of Seurat v4, which is robust to limited cell numbers.^12^ We used the “sctransform” modeling framework, which corrects counts for each gene in a cell by regressing out sequencing depth.^13^ The difference between G2M and S phase scores was regressed so that differences in cell cycle phase among proliferating cells were eliminated, while at the same time retaining signals separating cycling and non-cycling cells. For differential gene expression testing between clusters a Bonferroni-corrected *P*-value < .05 was considered significant. To perform gene set enrichment analysis, we first used the R package PRESTO which performs a fast Wilcoxon rank sum test on the Seurat clusters.^14^ We then tested the functional enrichment of Seurat clusters-of-interest using gene sets from MsigDB and the fgsea package.^15,16^ Visualization of pathway enrichment analysis was performed using g:Profiler to search for GO terms that were significantly enriched in a gene list before visualizing the results using EnrichmentMap in Cytoscape.^17,18^ Pathway gene sets with fewer than 3 genes and more than 350 genes were excluded in line with the default g:Profiler and Cytoscape workflows. For g:Profiler, a Benjamini-Hochberg false discovery rate adjusted *P*-value of < .05 was considered significant. Pseudotime analysis was conducted using Monocle 3.^19^ Dataset integrations were, in general, performed using standard workflows in Seurat v4, except when integrating malignant cell populations (Figure 5) where we observed strong blending of distinct malignant cell states. Instead, we used the fastMNN algorithm,^21^ which was found to preserve biological differences between cancer cell populations, while ensuring effective batch correction.^22^ Copy number alterations were inferred from scRNA-seq using inferCNV^23^ and visualized using Next Generation Clustered Heat Map.^24^

### Statistical Analysis

Statistical significance testing was performed using R.

## Results

We co-cultured malignant tumor spheroids of ONS-76 cells or DAOY cells, which harbor wild-type and mutant copies of *TP53*, respectively, with nonmalignant cerebellar organoids differentiated from human iPSC (Figure 1A; Supplementary Figure S1A). As controls, tumor cells were grown as monolayers or tumor spheroid monocultures and nonmalignant organoids were grown separately. These long-established cell lines were chosen because of their evolutionary divergence from patient tumors. Our approach was designed to enhance the detection of malignant cell states induced by the nonmalignant microenvironment, when comparing monocultures to the co-culture conditions. Using primary SHH-MB or PDX cultured cells could potentially mask such differences, given their closer resemblance to in vivo cell states, thus obscuring microenvironmental impacts on tumor cell properties. After 25 days in co-culture, cells were dissociated and processed for single-cell RNA sequencing (scRNA-seq) on the 10X Chromium platform. After filtering the dataset in accordance with our quality control thresholds we selected 13 534 cells for further analysis (Supplementary Figure S1B—E; Supplementary Table 2). We used Seurat to visualize the relationship between samples by clustering and projecting cells in a common UMAP space labeled according to their sample of origin (Supplementary Figure S1F, G). ONS-76 cells and DAOY cells cultured as monolayers or tumor spheroids formed well-demarcated clusters. In contrast, there was extensive overlap of co-cultured cells with the nonmalignant organoid control, which was anticipated given the large numbers of cells recovered belonging to organoid cellular subtypes. Only a handful of cells from the nonmalignant control organoid clustered with the tumor monoculture samples (Supplementary Figure S1F, G), confirming that the clustering reflected biological differences between the samples. By contrast, in the co-cultured samples, a larger proportion of cells clustered with the malignant monolayer and spheroid samples suggesting the latter cells were malignant cells (Supplementary Figure S1F, G).

### ONS-76 and DAOY Cells Cultured as Monolayers or Tumor Spheroids Were Enriched for Proliferative, Protein Biosynthetic, and Wound Healing Processes

We performed an integrated analysis of ONS-76 monolayer and tumor spheroid transcriptomes in Seurat^13^ and compared the transcriptional programs of malignant cells using functional enrichment analysis in the 2 conditions.^16^ ONS-76 cells grown as monolayers were enriched for Gene Ontology (GO) terms for cell cycle-associated genes (Supplementary Figure S2A and Table S3). In comparison, genes upregulated in tumor spheroids were associated with altered cell morphology and wound healing for example *IFITM3, IGFBP2, IGFBP5, STC1,* and *SPARC* (Supplementary Figure S2B—C and Table S3) consistent with an epithelial-to-mesenchymal transition state and tumor stroma production.^25^ Upon clustering of the ONS-76 tumor spheroid sample (Supplementary Figure S2D), expression of these genes was distributed over all clusters (Supplementary Figure S2E) supporting relative homogeneity of malignant cell states with mostly arbitrary distinctions between clusters, with 2 exceptions. The first consisted of a cluster of cycling cells, denoted ‘cluster 4’ that was marked by the expression of genes including *MKI67*, *MYBL2*, *BIRC5,* and *RRM2* consistent with a proliferative cell state (Supplementary Figure S2D, G). Although the same genes were expressed in the ONS-76 monolayer sample, they were not restricted to a single cluster (Supplementary Figure S2I). A second ONS-76 tumor spheroid cluster, ‘cluster 0’ expressed ribosomal genes and was enriched for protein synthesis pathways (Supplementary Figure S2D and Table S4), which is a feature common to many cancers.^26^ Less heterogeneity was evident in ONS-76 cells cultured as monolayers, which could be split into 2 clusters also distinguished by their relative enrichment for ribosomal genes and protein synthesis (Supplementary Figure S2H; Table S4). In comparison to ONS-76 tumor spheroids, expression of proliferation genes spanned both clusters, although the proliferation signal of individual cells was weaker (Supplementary Figure S2H, lower panel).

Broadly similar findings were observed upon analysis of DAOY cells cultured as monolayers and tumor spheroids. An integrated analysis of the cellular transcriptomes revealed protein synthesis gene sets as the only significant enrichment (Supplementary Table 5) in DAOY tumor spheroids. In common with ONS-76 cells, wound healing gene sets were also upregulated, but in DAOY monolayers rather than in DAOY tumor spheroids. Furthermore, there was a degree of enrichment in oncogenic signature gene sets that was statistically nonsignificant for transcriptional responses of cerebellar granule progenitors to activation by SHH, which acts as a mitogen for these cells (Supplementary Table 5).

Clustering of the DAOY tumor spheroid sample also revealed enrichment of protein synthesis and cell cycle-related pathways, respectively in 2 out of 5 DAOY tumor spheroid clusters (Supplementary Figure S2F; Table 5). Therefore, in tumor spheroids of both cell lines, those cells with a signature of enhanced proliferation were restricted to specific clusters in UMAP space, suggesting an association with a distinct cell state related to malignant cell proliferation. By contrast, DAOY monolayer cells that expressed a proliferative signature were spread diffusely across multiple clusters in common with ONS-76 monolayer cultured cells (Supplementary Figure S2J). Although DAOY monolayer cells formed more clusters than ONS-76 cells (6 in DAOY monolayers compared to 2 in the corresponding ONS-76 sample), only 3 out of 6 of the former clusters displayed significant gene set enrichment. Of these, 2 clusters were enriched for protein synthesis pathways and the remaining cluster was only significantly enriched for cell adhesion-related gene sets, which is a hallmark of cancer (Supplementary Table 5).^27^ Assessment of the maturity of malignant cells from tumor spheroid and monolayer samples of both cell lines revealed they expressed the granule cell markers, *MEIS1,*^28^*MEIS2*^29^ and *JKAMP*^30^ (Supplementary Figure S2K). However, *CNTN1* which marks differentiating granule cells^31^ was selectively expressed only in ONS-76 samples, suggesting the maturation of DAOY cells was relatively impaired (Supplementary Figure S2K).

### ONS-76 Cells in Co-culture, But Not In Monolayers or Tumor Spheroids Expressed *NEUROD1*

To determine whether malignant cells grown in a nonmalignant organoid microenvironment led to altered tumor cell phenotypes, we clustered the ONS-76 sample in co-culture and plotted the UMAP projection, comparing the clusters obtained with those in the nonmalignant control organoid (Figure 1B; Supplementary Figure S3A). The manual annotations of nonmalignant cells were highly similar to our earlier annotations of day 90 organoids.^32^ None of the clusters of malignant cells in co-culture were identified as having a match in the control organoid. We additionally confirmed the robustness of our classification of malignant versus nonmalignant cells in co-culture by analysis of copy number alterations (Supplementary Figure S3B).

In the control organoid, granule neurons could be defined by their co-expression of *NEUROD1*, *RTN1,* and *RBFOX3* (Figure 1C).^30^ In the co-culture condition also, a cluster of cells co-expressing these genes could be identified consistent with nonmalignant granule neurons (Figure 1D). Additionally, a distinct cell cluster located adjacent to nonmalignant granule neurons in UMAP space could be distinguished from the latter cells by their expression of *NEUROD1* only, which we labeled ‘ONS76_cluster_3’ (Figure 1D; Supplementary Figure S3C; Supplementary Table 6). Furthermore, upon computational integration of ONS-76 samples malignant *NEUROD1*^+^ cells were absent in ONS-76 monolayers and tumor spheroids (Figure 1E) and only very few were present in the DAOY samples (Figure 1E, Supplementary Figure S3D).

Although *NEUROD1* marks post-mitotic granule neurons in mouse,^33^ in humans this gene is also expressed in cycling granule progenitors.^34^ Gene set enrichment analysis of ‘ONS76_cluster_3’ revealed GO terms associated with cycling cells (Figure 1F; Supplementary Figure S3E). By contrast, the cluster annotated as ‘Granule neurons’ in the nonmalignant organoid in co-culture and in the control organoid were enriched for terms associated with differentiated neurons (Figure 1G; Supplementary Figure S3F—H). To validate these findings, we computationally examined the cell cycle phase associated with *NEUROD1* expression in nonmalignant and malignant cells.^13^ In the cluster annotated as ‘Granule neurons’ in the control organoid (Figure 1C), the fraction of *NEUROD1*^+^ cells that were in the G1 phase was 0.60, in the G2/M phase the fraction was 0.17 and the S phase fraction was 0.23. However, in ONS-76 cells in co-culture the fraction of *NEUROD1*^+^ cells in “ONS76_cluster_3” in G1 phase was smaller (0.38), and the fraction of cells in G2/M and S phases was higher (0.30 and 0.32, respectively; *P* = 5.925 × 10^-12^ Chi-squared test). Therefore, ONS-76 cells activated *NEUROD1,* a key molecular determinant of granule cell identity only when embedded in a more physiological microenvironment, but its expression was less tightly coupled to other cellular processes that imparted a differentiated neuronal identity. By comparison, analysis of patient SHH-MB scRNA-seq public datasets showed that they also expressed *NEUROD1* (Supplementary Figure S4A-E)^20,35^ and that for one of the datasets^20^ most of these cells were in the G1 phase (range 0.59–0.69). Consistent with the maturation of malignant cells, patient tumors also expressed *RBFOX3* and *RTN1* (Supplementary Figure S4A—C, E). Given the absence of *NEUROD1* upregulation in DAOY cells in co-culture, we analyzed its expression in 2 *TP53* mutant SHH-MB in a second public MB scRNA-seq dataset to test for an association between the presence of the mutation and *NEUROD1* expression in vivo.^35^ The tumors varied widely in their expression of *NEUROD1* displaying both elevated and minimal expression (Supplementary Figure S4D). Therefore, the absence of *NEUROD1* expression in *TP53* mutant DAOY cells in tumor spheroid-organoid co-culture was not inconsistent with in vivo findings.

A second, smaller cluster of ONS-76 tumor cells in co-culture, ‘ONS76_cluster_10’ was identified by differential upregulation of *HOXB2* and *POU3F2*, in common with multiple non-CNS cancers including leukemia, lung, and breast cancer (Supplementary Figure S5A; Supplementary Table 6).^36,37^ Cells in this cluster also expressed *MEIS1*, which is expressed by differentiating granule cells^38^ and *HOXB-AS1*, which is a member of a large family of non-coding transcripts that are abnormally expressed in a wide range of cancers.^39^ Consistent with the expression of *MEIS1*, gene set enrichment analysis suggested this cluster contained mature cells (Supplementary Figure S5B, C). Overall, subpopulations of malignant cells expressed in vivo markers of differentiation when they were surrounded by the more physiologic microenvironment of the organoid.

### A Subpopulation of DAOY Cells in Co-culture Expressed Multiple CSC-Like Markers

**Figure 2.**
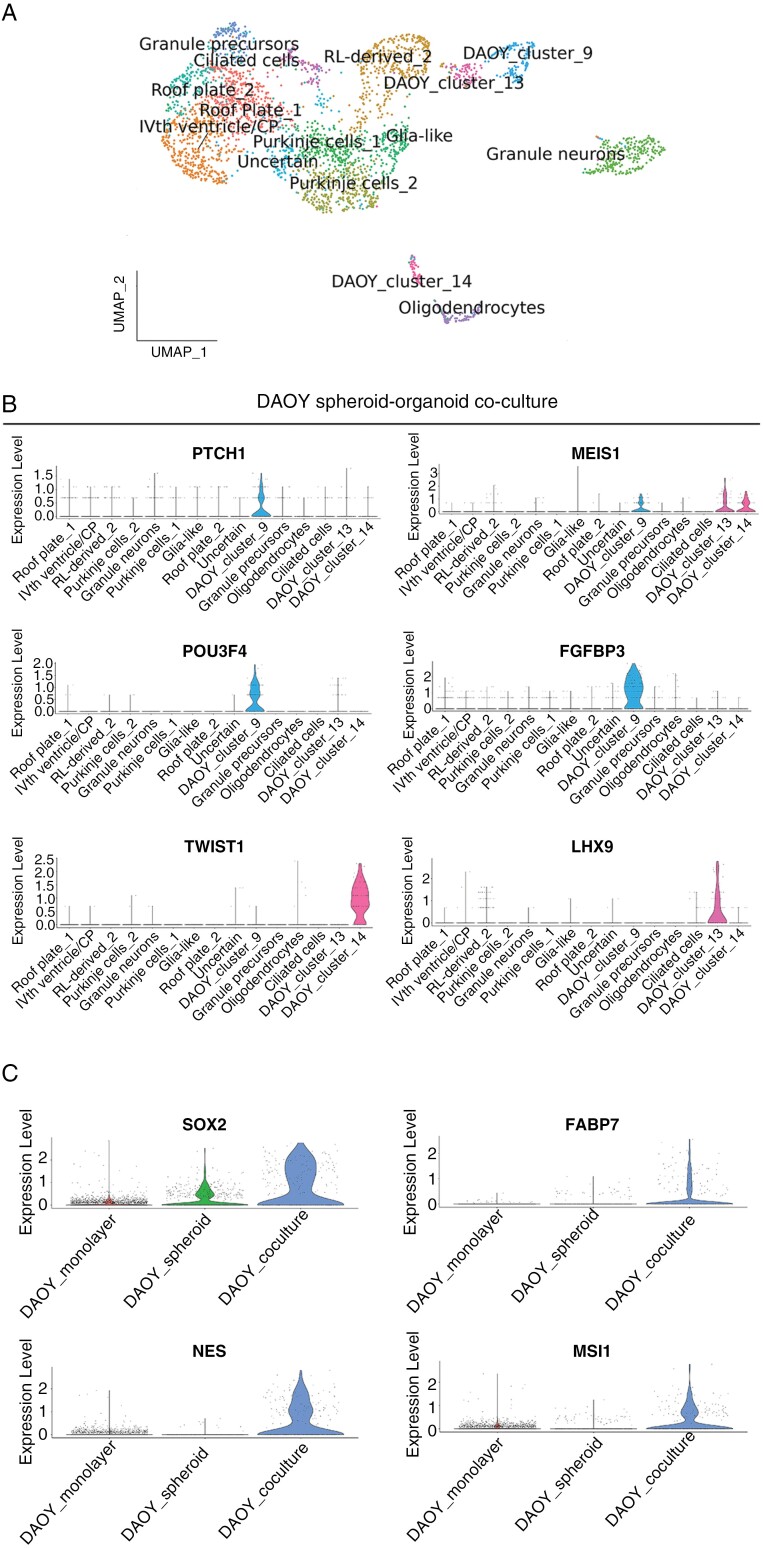
Upregulation of neural stem cell markers in DAOY malignant cells in co-culture. (A) UMAP visualization of single-cell transcriptomes in DAOY tumor spheroid-organoid co-cultures shows 3 malignant clusters annotated as ‘DAOY_cluster_9’, ‘DAOY_cluster_13’ and ‘DAOY_cluster_14.’ (B) Violin plots showing expression of unique markers of malignant DAOY clusters in co-culture. (C) Violin plots of expression of the indicated neural stem cell markers in the DAOY monolayer culture, DAOY tumor spheroid, and DAOY cells in tumor spheroid-organoid co-culture.

The analysis of the DAOY tumor spheroid-organoid co-culture sample resulted in the clustering of DAOY tumor cells into 3 distinct groups in UMAP space designated as ‘DAOY_cluster_9’, ‘DAOY_cluster_13’, and ‘DAOY_cluster_14’ (Figure 2A). Highly distinctive, differential expression of specific markers of these putative malignant clusters was apparent, exemplified by *PTCH1, MEIS1, POU3F4, FGFBP3, TWIST1,* and *LHX9* that displayed low or no expression in nonmalignant cells in co-culture and in the control organoid (Figure 2B, Supplementary Figure S5D and Table S6). Additional evidence that cells in these clusters were malignant was obtained by analysis of copy number alterations (Supplementary Figure S3B). Noting that *POU3F4* and *FGFBP3* have been linked to a neural stem cell (NSC) identity,^40,41^ we compared DAOY cells in co-culture with the DAOY tumor spheroid sample for expression of NSC markers (Supplementary Figure S5E). Multiple NSC markers were upregulated in DAOY cells in co-culture including *SOX2*, *NES*, *FABP7,* and *MSI1* (Figure 2C). A similar trend was observed for ONS-76 cells in the 3 conditions, although the upregulation of these NSC markers in ONS-76 cells in co-culture was not as great (Supplementary Figure S5F).

**Figure 3.**
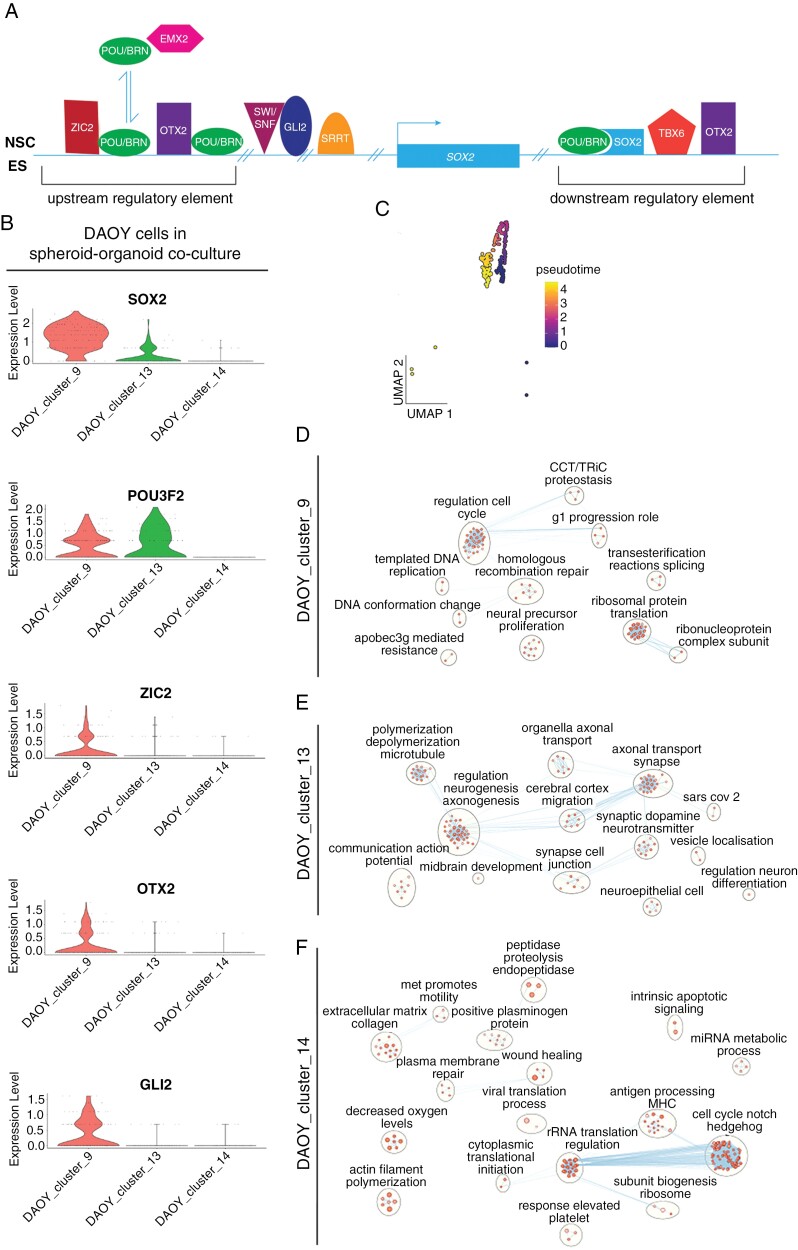
Multiple factors known to regulate the expression of *SOX2* in neural stem cells and ES cells (ESC) are also expressed in a subpopulation of DAOY cells in co-culture. (A) Schematic of the *SOX2* regulatory network in neural stem cells and ESC (adapted from Bertolini et al.^43^). (B) Violin plots of expression of the *SOX2* regulatory network in ‘DAOY_cluster_9’ cells. (C) Pseudotime trajectory of cells in ‘DAOY_cluster_9’. (D) Enrichment of GO biological pathway terms in ‘DAOY_cluster_9’ visualized in Cytoscape.^18^ (E) Enrichment of GO biological pathway terms in ‘DAOY_cluster_13’ visualized in Cytoscape. (F) Enrichment of GO biological pathway terms in ‘DAOY_cluster_14’ visualized in Cytoscape. *TBX6* was not expressed in malignant cells.

The upregulation of NSC markers in malignant cells in our co-culture model is reminiscent of mouse models of SHH-MB, in which persistent SOX2-expressing granule cells designated as CSC drive tumor formation and relapse.^6,42^ We therefore focused our analysis on further characterizing the transcriptional profile of these cells. A more granular analysis at the level of individual malignant clusters revealed striking expression of factors known to regulate *SOX2* (Figure 3A, B).^43^ Only a single DAOY cluster in co-culture, ‘DAOY_cluster_9’ expressed multiple members of the *SOX2* regulatory network consisting of *POU3F2*, *ZIC2*, *OTX2*, *GLI2,* and *SOX2* itself (Figure 3B). This combinatorial pattern of gene expression was not observed in ONS-76 cells in co-culture (Supplementary Figure S6A), nor in tumor spheroid or monolayer samples of either cell line (Supplementary Figure S6B), or in nonmalignant organoid cells (Supplementary Figure S6C). The co-expression of these markers in subpopulations of DAOY cells in co-culture suggested these cells adopted a new cell state in the presence of a nonmalignant microenvironment.

We hypothesized there could be an ontological relationship between the distinct DAOY cell states identified in co-culture. In particular, the UMAP plot of DAOY cells in co-culture (Figure 2A) suggested ‘DAOY_cluster_9’ and ‘DAOY_cluster_13’ cells occupied opposite ends of a single trajectory. A pseudotime analysis supported the notion that ‘DAOY_cluster_9’ could undergo a progressive transition to a ‘DAOY_cluster_13’ cell state (Figure 3C). Functional enrichment analysis was consistent with the pseudotime analysis and showed that ‘DAOY_cluster_9’ cells were enriched for GO terms associated with a proliferative state (Figure 3D; Supplementary Table 7), whereas ‘DAOY_cluster_13’ was enriched for neuronal differentiation terms (Figure 3E and Supplementary Table 7). The last cluster, ‘DAOY_cluster_14’ was transcriptionally less similar to the other DAOY clusters (Figure 2A). Enriched pathways for this cluster related to Notch and Hedgehog signaling, and translation regulation and included extracellular matrix composition and wound healing gene sets in common with tumor cells cultured in isolation (Figure 3F; Supplementary Table 7).

### CSC-like Cells With a Molecular Profile Resembling DAOY Tumor Cells in Co-culture Are Present in Patient SHH-MB

**Figure 4.**
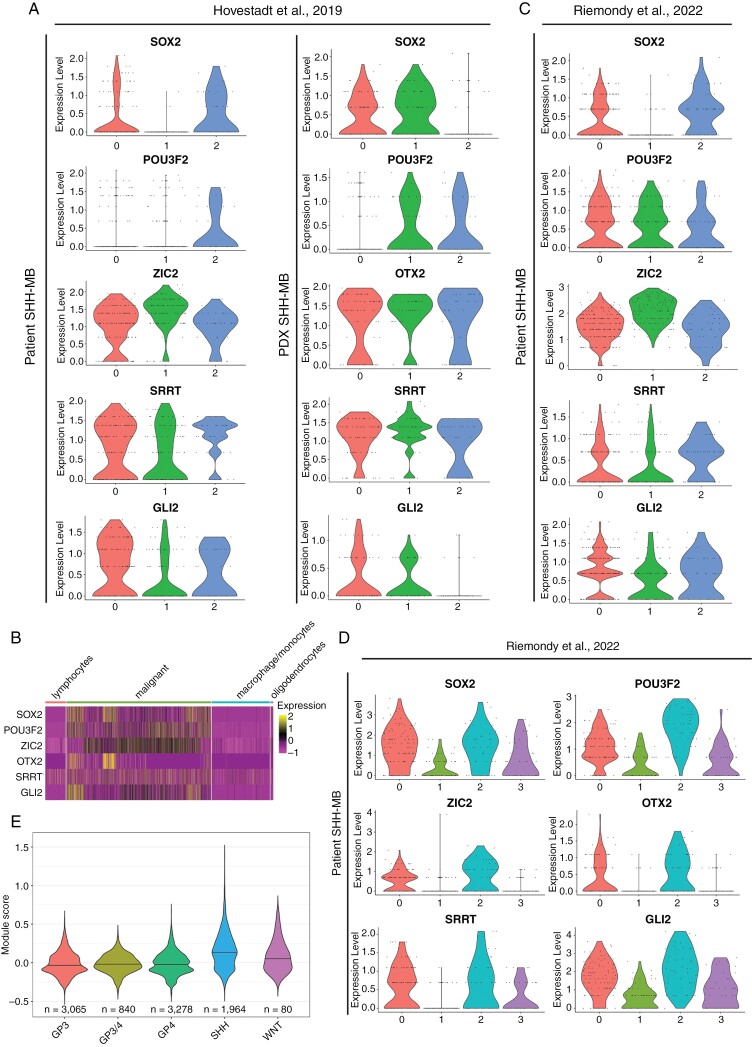
Expression of the *SOX2* regulatory network in patient SHH-MB datasets sequenced at single-cell level. (A) Violin plots of the expression of the *SOX2* regulatory network in 1 of the 3 patient SHH-MB (tumor ID: SJ454) and in a SHH-MB PDX tumor model (tumor ID: RMCB18) from the same dataset.^20^ (B) Heatmap of expression of the *SOX2* regulatory network in malignant cells and in cells of the microenvironment in a second patient SHH-MB dataset.^35^ Cell labels are retained from the original publication. (C) Expression of the *SOX2* regulatory network in 2 of 3 UMAP clusters in a patient tumor (tumor ID: 1235) from the same dataset.^35^ (D) *SOX2* regulatory network expression in another tumor (tumor ID: 801, *TP53* mutant) from the same dataset.^35^ (E) Violin plots of cell scores for the *SOX2* regulatory network in all medulloblastoma subtypes. Only malignant cells are included.^35^*TBX6* was not expressed in malignant cells in either dataset.

We analyzed the expression of the *SOX2* regulatory network in a public scRNA-seq dataset of patient SHH-MB.^20^ Upon clustering tumor cells in Seurat^13^ a single cluster in 1 of the 3 SHH-MB tumors expressed a highly similar network in which *OTX2* was replaced by expression of another member of the regulatory network, *SRRT* (Figures 3A and 4A).^43^ Furthermore, in 1 of the 2 PDX models of SHH-MB from the same dataset,^20^ a single cluster expressed a common network, consisting of *POU3F2*, *OTX2*, *SRRT*, *GLI2,* and *SOX2* (Figure 4A). Given these differences, and to determine the prevalence and identity of cells expressing a signature of a *SOX2* regulatory network, we analyzed a larger dataset of patient SHH-MB.^35^ Initial analysis of the data showed upregulation of all the aforementioned genes in malignant cells compared to the nonmalignant microenvironment (Figure 4B). By contrast, co-expression of these genes was weaker in other MB subtypes (Supplementary Figure S7A). Clustering the individual SHH-MB tumors showed that in 7/8 samples 2 to 4 clusters emerged per tumor. One or more of these clusters expressed a *SOX2* regulatory network that was highly similar to the regulatory network in DAOY cells in co-culture (Figure 4C, D; Supplementary Figure S7B—G). The proportion of malignant cells expressing this signature ranged from 4.2% to 20.3% (median 8.8%) in this dataset.^35^ Quantification of the gene signature across MB subtypes showed enrichment in SHH-MB tumors (Figure 4E). Furthermore, the expression of the gene signature was unrelated to *TP53* mutation status (*n* = 2 *TP53* mutant SHH-MB tumors) and was present in both *TP53* wild-type (Figure 4A and C and Supplementary Figure S7B—F) and *TP53* mutant (Figure 4D and Supplementary Figure S5G) patient tumors. These data confirmed that the patterns of gene expression in DAOY cells in co-culture were not simply an artifact related to our culture conditions, and instead suggested that DAOY cells mirrored a CSC-like state found in vivo.

### Greater Transcriptional Similarity of DAOY and ONS-76 Cells in Co-culture to Patient SHH-MB

**Figure 5.**
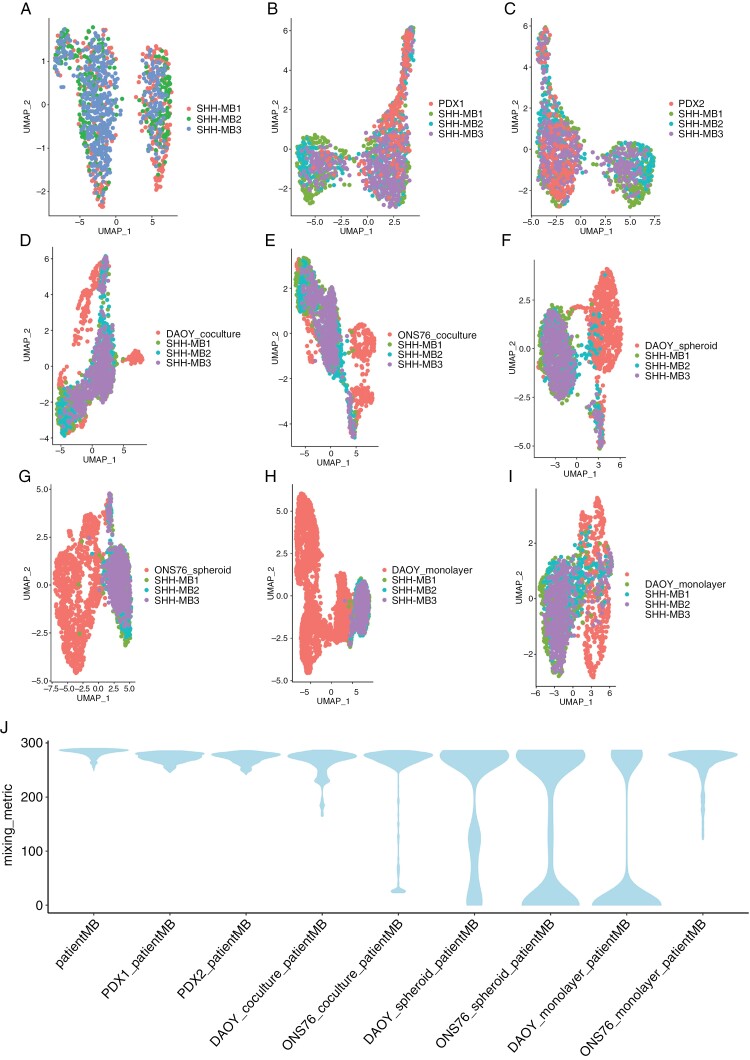
The co-culture tumor models recapitulate SHH-MB patient tumors better than conventional in vitro models. (A) UMAP visualization of 3 SHH-MB patient tumors. (B) Integration and UMAP visualization of cells from a PDX SHH-MB tumor (PDX1) with the same 3 SHH-MB patient tumors as in A. (C) Integration and UMAP visualization of cells from a second PDX SHH-MB tumor (PDX2) with the 3 SHH-MB patient tumors. (D) Integration and UMAP visualization of DAOY cells in tumor spheroid-organoid co-culture with the 3 SHH-MB patient tumors. (E) Integration and UMAP visualization of ONS-76 cells in tumor spheroid-organoid co-culture with the 3 patient SHH-MB tumors. (F) Integration and UMAP visualization of DAOY tumor spheroid monoculture cells with the 3 patient SHH-MB tumors. (G) Integration and UMAP visualization of ONS-76 tumor spheroid monoculture cells with the 3 patient SHH-MB tumors. (H) Integration and UMAP visualization of DAOY monolayer cells with the 3 patient SHH-MB tumors. (I) Integration and UMAP visualization of ONS-76 monolayer cells with the 3 patient SHH-MB tumors. (J) Violin plots of mixing metric scores of the integrations shown in A to I. Patient and PDX tumor datasets were obtained from the published study by Hovestadt et al.^20^ Only malignant cells were used for scoring.

Encouraged by the transcriptional evidence suggesting tumor heterogeneity resembling SHH-MB in vivo in our organoid co-culture model, we assessed the transcriptional similarity of our model to patient tumors and PDX models. Therefore, we compared the computational integration of DAOY and ONS-76 tumor cells in co-culture and PDX models with patient SHH-MB tumors. We did not use canonical correlation methodology implemented in Seurat for this part of the analysis because this algorithm excessively blends distinct cancer cell states.^22^ Instead, we employed the fastMNN algorithm,^44^ which performed well in an earlier benchmarking study of tumor datasets^22^ to integrate 3 public SHH-MB patient single-cell datasets.^20^ Upon projection in UMAP space 2 major clusters emerged to which all 3 tumors contributed (Figure 5A). Two SHH-MB PDX tumours^20^ blended well with the patient tumors but showed greater mixing with 1 of the 2 major clusters, suggesting these PDX models did not recapitulate the full extent of transcriptional diversity of the patient tumors (Figure 5B, C). Whereas DAOY and ONS-76 cells in co-culture, as expected, were less similar to patient tumors (Figure 5D, E), in comparison with tumor spheroids of either cell line and the DAOY monolayer sample far greater similarity was evident (Figure 5F—H). We also computed a “mixing metric” to quantify how similar these datasets were after integration (Figure 5J and Supplementary Table 8).^45^ Patient tumors scored the highest on this metric, followed closely by PDX tumors. Strikingly, our tumor spheroid-organoid co-culture models also performed well, in contrast to tumor spheroid monocultures. In the ONS-76 monolayer cultures, transcriptional mixing with patient tumors was evident, as demonstrated by the UMAP plot (Figure 5I) and the mixing metric plot (Figure 5J). While these results indicate that the ONS-76 monolayer shares some transcriptional features with patient tumor cells, the ONS-76 co-culture model exhibits a more profound phenotypic similarity to patient SHH-MB tumors. This closer resemblance is particularly highlighted by the presence of *NEUROD1*-expressing cells in the co-cultures, which were absent in both the ONS-76 monolayer and spheroid cultures (Figure 1E). These findings underscore the enhanced capability of the co-culture model to more accurately replicate the cellular heterogeneity of SHH-MB in vivo, thus supporting its application as a superior in vitro model for studying this disease.

## Discussion

Human cerebellar organoid-based in vitro models have the potential to recapitulate patient MB more faithfully compared to conventional in vitro models.^46^ Here, we tested the importance of the nonmalignant microenvironment in regulating malignant cell states by comparing the phenotypes of 2 long-established SHH-MB lines grown in co-culture or conventionally. Compared to conventional in vitro models, we detected greater maturity of malignant cells in co-culture. In particular, a subpopulation of ONS-76 cells activated the expression of *NEUROD1*, a canonical marker of granule cells, only upon co-culture. Additionally, a subpopulation of DAOY cells in co-culture expressed CSC-like markers, and pseudotime and functional enrichment analyses were concordant attesting to their capacity for maturation into cells with neuronal features. The expression of CSC-like markers and upregulation of neuronal differentiation genes by malignant cells in co-culture revealed the emergence of cell states that were not observed in tumor cells cultured in isolation, under our conditions. Given that similar cell states are found in mouse models, PDX models, and upon patient tumor molecular profiling,^6,20,42,47^ we further demonstrated the convergence of 2 SHH-MB cell lines towards in vivo cellular phenotypes. Therefore, the microenvironment can non-cell autonomously regulate SHH-MB cell states. Moreover, our computational analysis effectively addresses the challenge of the high nonmalignant to malignant cell ratio in our co-cultures. The resilience of Seurat to cell downsampling made the identification of patient tumor-relevant cell states in our co-culture model possible.^12^

Malignant cell phenotypes in tumor spheroid-organoid co-culture were similar to those seen in vivo in patient tumors and PDX models (Figure 1D and E, 3B, 4 and, 5) thereby confirming the validity of our model in recapitulating patient tumors better than conventional in vitro models. Furthermore, our findings suggest that the consensus view that these cell lines are poorly representative of patient tumors does not hold true when these malignant cell lines are cultured in a more physiological context. The emergence of new cell states in these long-established SHH-MB cell lines that were not evident in monolayer or tumor spheroid monoculture conditions was striking, more so given the relatively short duration (25 days) of co-culture. The immediate cause of these rapid phenotypic changes is likely to be transcriptional plasticity, reflecting functional intra-tumor heterogeneity that is shared by diverse malignant tumors.^48^

Malignant granule cells can differentiate in patient SHH-MB and in mouse models of this disease.^20,35,47,49,50^ Interestingly, the activation of *NEUROD1* in SHH-MB patient samples showed no dependency on *TP53* mutational status (Supplementary Figure S4D), suggesting that differentiation processes can still be initiated in the presence of somatic *TP53* mutations.^51^*NEUROD1* was upregulated in a subpopulation of ONS-76 cells in tumor spheroids in co-culture with organoids compared to tumor monocultures (Figure 1E). In both healthy and malignant cells, expression of *NEUROD1* was associated with actively cycling cells and cells with neuronal features (Figure 1F and G and Supplementary Figure S3E—H). Interestingly, however, in the malignant subpopulation, upregulation of *NEUROD1* was not as tightly coupled with neuronal differentiation compared to nonmalignant granule cells. These differences could reflect the divergence of the properties of ONS-76 cells over an extended period in vitro, favoring the survival of cycling cells over more differentiated cells. Recent analysis of human granule cell ontogeny has demonstrated that, in contrast to mouse, these cells express *NEUROD1* during their cycling phase.^34^ The expression of this gene in a smaller proportion of nonmalignant cycling granule precursors is consistent with the latter finding and further validates our cerebellar organoid model.

Our analysis showed that the DAOY CSC-like cells present in co-culture were enriched for gene sets related to the cell cycle, which suggested they were proliferative (Figure 3D). Moreover, closely similar or equivalent cell states as defined by expression of the *SOX2* regulatory network were present in a majority of samples across 2 published scRNA-seq datasets that spanned infant to adult subtypes (Figure 4 and Supplementary Figure S7B—G).^20,35^ Reasons for the differences in the expressed members of the *SOX2* regulatory network in CSC-like subpopulations are unclear. Differences could relate to the redundancy of distinct *SOX2* regulators or reflect the divergence of functional states within CSC-like populations, associated with, for example, different proliferation rates and sensitivity to therapy. The traditional view is that CSCs are rare, slow-cycling cells that are relatively quiescent compared to other malignant cells, and such tumor-initiating cells were identified in a SHH-MB mouse model.^6,42^ The proportion of CSC-like cells in the scRNA-seq dataset we analysed^35^ was up to ~20% with a median close to 10%, suggesting these cells might be more common than previously thought. Moreover, in another brain tumor, glioma, stem cells have also been reported to be proliferative, supporting the view that CSC states are non-uniform.^4^ Future functional studies are needed to determine if the SHH-MB cells with the signature we identified meet the standard for stemness in in vivo limiting dilution assays. Pseudotime and functional enrichment analyses were consistent and suggested these cells underwent a maturation process, associated with a continuum of cell states that led to the acquisition of neuron-like features (Figure 3C—E). Furthermore, we discounted the possibility that the emergence of a CSC-like state in DAOY cells was caused by the *TP53* mutation because of the prevalence of a CSC-like state in patient SHH-MB analyzed here that were wild type for *TP53* (Figure 4A, C and Supplementary Figure S7B—F) and the expression of NSC markers in ONS-76 cells in tumor spheroid-organoid co-culture (Supplementary Figure S5F).

In our co-culture model, single or multiple constituents of the nonmalignant microenvironment could induce the emergent phenotypic states of SHH-MB cells. Regulatory interactions between malignant and nonmalignant components that exploit local signaling networks are a well-established mechanism for tumor cells to gain a growth advantage.^52^ Microenvironmental factors implicated in these signaling networks in vivo include stromal cells, blood and lymphatic vessels, immune-inflammatory cells, and extracellular matrix (ECM). Of these, ECM is the only one of these factors represented in our co-culture model and has a well-recognized role in tumor progression.^53^ Whether other components of the microenvironment in our model, namely progenitors, neurons, glia, roof plate, or choroid plexus can produce malignant cell state-reprogramming signals is unclear. Identification of this factor(s) in the future could open new therapeutic options.

Our co-culture model provides a platform for advances in the biological underpinnings of medulloblastoma and has translational applications. It should be possible to leverage the scRNA-seq data to dissect the signaling pathways driving tumor-microenvironment interactions,^54^ potentially revealing novel therapeutic targets. To determine the utility of our model as a pre-clinical platform, it will be important to test whether cerebellar organoids can replicate the in vivo environment of PDX tumors orthotopically grafted into the mouse cerebellum. The model can be further enhanced by incorporating iPSC-derived microglia immune components to better mimic the in vivo tumor microenvironment. Additionally, this system facilitates the testing of therapies not only on tumor cells but also on the surrounding microenvironment, paving the way for strategies that prioritize both tumor eradication and neuronal protection. These applications highlight the versatility of our co-culture method in addressing key biological questions and informing the development of more effective, less neurotoxic treatments for medulloblastoma.

## Supplementary Material

vdae218_suppl_Supplementary_Tables_S1-S8_Figures_S1-S7

## Data Availability

scRNA-seq data have been deposited in the National Centre for Biotechnology Information Gene Expression Omnibus (GEO) database and are publicly accessible through GEO accession number GSE254917 (https://www.ncbi.nlm.nih.gov/geo/query/acc.cgi?acc=GSE254917). Analysis code is available here or on Zenodo.
