# Supplementary material for: Sonic hedgehog medulloblastoma cells in co-culture with cerebellar organoids converge towards in vivo malignant cell states
Source: Neurooncol Adv. 2024 Dec 13;7(1):vdae218. doi: 10.1093/noajnl/vdae218 (PMC11783571; doi:10.1093/noajnl/vdae218)
Supplement: vdae218_suppl_Supplementary_Tables_S1-S8_Figures_S1-S7 [file vdae218_suppl_supplementary_tables_s1-s8_figures_s1-s7.zip › Supplementary_tables_titles.docx]

**Supplementary Tables**

**Supplementary Table 1**. Authentication of DAOY-GFP and ONS-76-Luc-GFP cell lines by STR profiling.

**Supplementary Table 2**. Sample cell numbers.

**Supplementary Table 3**. Genes expressed in ONS-76 monolayer and ONS-76 tumour spheroid cells.

**Supplementary Table 4**. Gene set Enrichment for GO Biological Process terms in ONS-76 tumour spheroid cluster 0, ONS-76 tumour spheroid cluster 4 and ONS-76 monolayer cluster 0.

**Supplementary Table 5**. Gene set enrichment for GO biological process terms and for C6 oncogenic signature gene sets in integrated DAOY monolayer and tumour spheroid samples, and in individual UMAP clusters of DAOY monolayer and tumour spheroid samples.

**Supplementary Table 6**. Markers of ONS-76 and DAOY malignant cell clusters in tumour spheroid-organoid co-culture.

**Supplementary Table 7**. g:Profiler enriched pathways in DAOY cells in tumour spheroid-organoid co-culture.

**Supplementary Table 8**. Mixing metric summary statistics quantifying the extent of intermixing of patient SHH-medulloblastoma samples with other samples.
