# Supplementary material for: Sonic hedgehog medulloblastoma cells in co-culture with cerebellar organoids converge towards in vivo malignant cell states
Source: Neurooncol Adv. 2024 Dec 13;7(1):vdae218. doi: 10.1093/noajnl/vdae218 (PMC11783571; doi:10.1093/noajnl/vdae218)
Supplement: vdae218_suppl_Supplementary_Tables_S1-S8_Figures_S1-S7 [file vdae218_suppl_supplementary_tables_s1-s8_figures_s1-s7.zip › Supplementary_Figures.pdf]

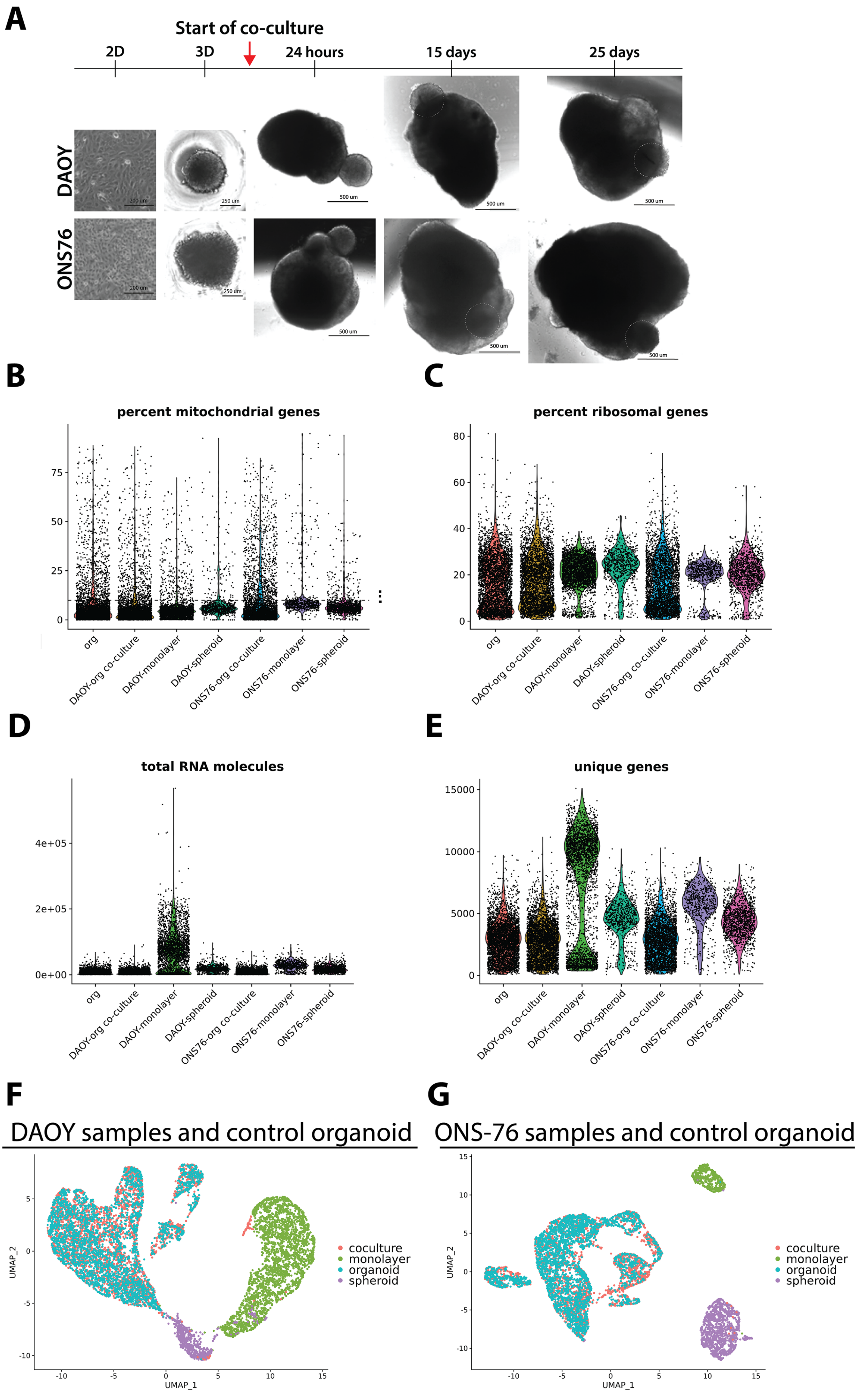
A
Start of co-culture
2D
3D
24 hours
15 days
25 days
DAOY
ONS76
B
percent mitochondrial genes
C
percent ribosomal genes
org
DAOY-org co-culture
DAOY-monolayer
DAOY-spheroid
ONS76-org co-culture
ONS76-monolayer
ONS76-spheroid
D
total RNA molecules
E
unique genes
F
DAOY samples and control organoid
G
ONS-76 samples and control organoid
coculture
monolayer
organoid
spheroid
UMAP_1
UMAP_2

Supplementary Figure S1. QC metrics on samples used in this study. (A) Bright-field images of DAOY and ONS-76 monolayers, tumour spheroids and co-cultures with non-malignant cerebellar organoids. (B) Percent mitochondrial genes. (C) Percent ribosomal genes. (D) Total number of RNA molecules. (E) Number of unique genes. (F) UMAP visualisation of DAOY monolayer, spheroid, co-culture and control non-malignant cerebellar organoid samples. (G) UMAP visualisation of ONS-76 monolayer, spheroid, co-culture and control non-malignant cerebellar organoid samples.

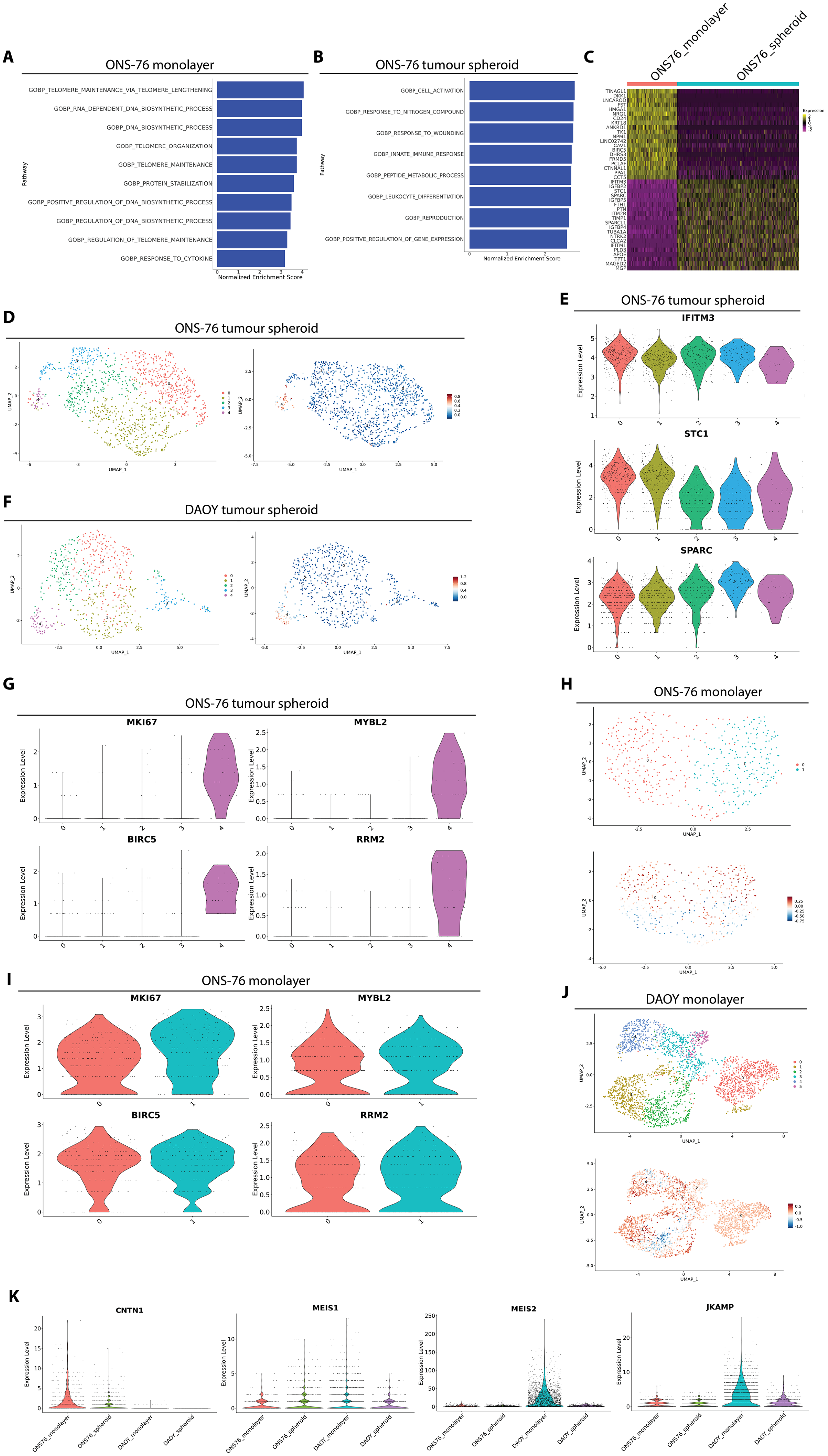
A
ONS-76 monolayer
GOBP_TELOMERE_MAINTENANCE_VIA_TELOMERE_LENGTHENING
GOBP_RNA_DEPENDENT_DNA_BIOSYNTHETIC_PROCESS
GOBP_DNA_BIOSYNTHETIC_PROCESS
GOBP_TELOMERE_ORGANIZATION
GOBP_TELOMERE_MAINTENANCE
GOBP_PROTEIN_STABILIZATION
GOBP_POSITIVE_REGULATION_OF_DNA_BIOSYNTHETIC_PROCESS
GOBP_REGULATION_OF_DNA_BIOSYNTHETIC_PROCESS
GOBP_REGULATION_OF_TELOMERE_MAINTENANCE
GOBP_RESPONSE_TO_CYTOKINE
Pathway
Normalized Enrichment Score
B
ONS-76 tumour spheroid
GOBP_CELL_ACTIVATION
GOBP_RESPONSE_TO_NITROGEN_COMPOUND
GOBP_RESPONSE_TO_WOUNDING
GOBP_INNATE_IMMUNE_RESPONSE
GOBP_PEPTIDE_METABOLIC_PROCESS
GOBP_LEUKOCYTE_DIFFERENTIATION
GOBP_REPRODUCTION
GOBP_POSITIVE_REGULATION_OF_GENE_EXPRESSION
Pathway
Normalized Enrichment Score
C
ONS76_monolayer
ONS76_spheroid
Expression
D
ONS-76 tumour spheroid
UMAP_1
UMAP_2
E
ONS-76 tumour spheroid
IFITM3
STC1
SPARC
Expression Level
F
DAOY tumour spheroid
UMAP_1
UMAP_2
G
ONS-76 tumour spheroid
MKI67
MYBL2
BIRC5
RRM2
Expression Level
H
ONS-76 monolayer
UMAP_1
UMAP_2
I
ONS-76 monolayer
MKI67
MYBL2
BIRC5
RRM2
Expression Level
J
DAOY monolayer
UMAP_1
UMAP_2
K
CNTN1
MEIS1
MEIS2
JKAMP
Expression Level
ONS76_monolayer
ONS76_spheroid
DAOY_monolayer
DAOY_spheroid

Supplementary Figure S2. Gene expression in ONS-76 and DAOY monolayers and tumour spheroids. (A) fgsea geneset enrichment of upregulated GO biological pathway terms evaluated by normalised enrichment scores in ONS-76 monolayer cells. (B) fgsea geneset enrichment of upregulated GO biological pathway terms evaluated by normalised enrichment scores in ONS-76 tumour spheroids. (C) Heatmap of top twenty differentially expressed genes in ONS-76 monolayer and tumour spheroid samples. (D) UMAP visualisation of ONS-76 tumour spheroid single cell transcriptomes identified five transcriptional cell clusters, 0 – 4 (left panel). Cell proliferation scores are superimposed on the UMAP visualisations (right panel). (E) Violin plots of expression of the indicated genes in transcriptional clusters of ONS-76 spheroids that are associated with epithelial mesenchymal transition and stroma production. (F) UMAP visualisation of single cell transcriptomes of DAOY tumour spheroids identified five transcriptional cell clusters, 0 – 4 (left panel). Cell proliferation scores are superimposed on the UMAP visualisations (right panel). (G) Violin plots of the expression of the indicated genes in ONS-76 tumour spheroid clusters. (H) UMAP visualisation of single cell transcriptomes of ONS-76 monolayer cells identified two transcriptional clusters, 0 – 1 (upper panel). Cell proliferation scores are superimposed on the UMAP visualisations (lower panel). (I) Violin plots of gene expression in ONS-76 monolayer transcriptional clusters. (J) UMAP visualisation of single cell transcriptomes of ONS-76 monolayer cells identified six transcriptional clusters, 0 – 5 (upper panel). Cell proliferation scores are superimposed on the UMAP visualisations (lower panel). (K) Expression of differentiation markers in ONS-76 and DAOY monolayer and tumour spheroid samples.

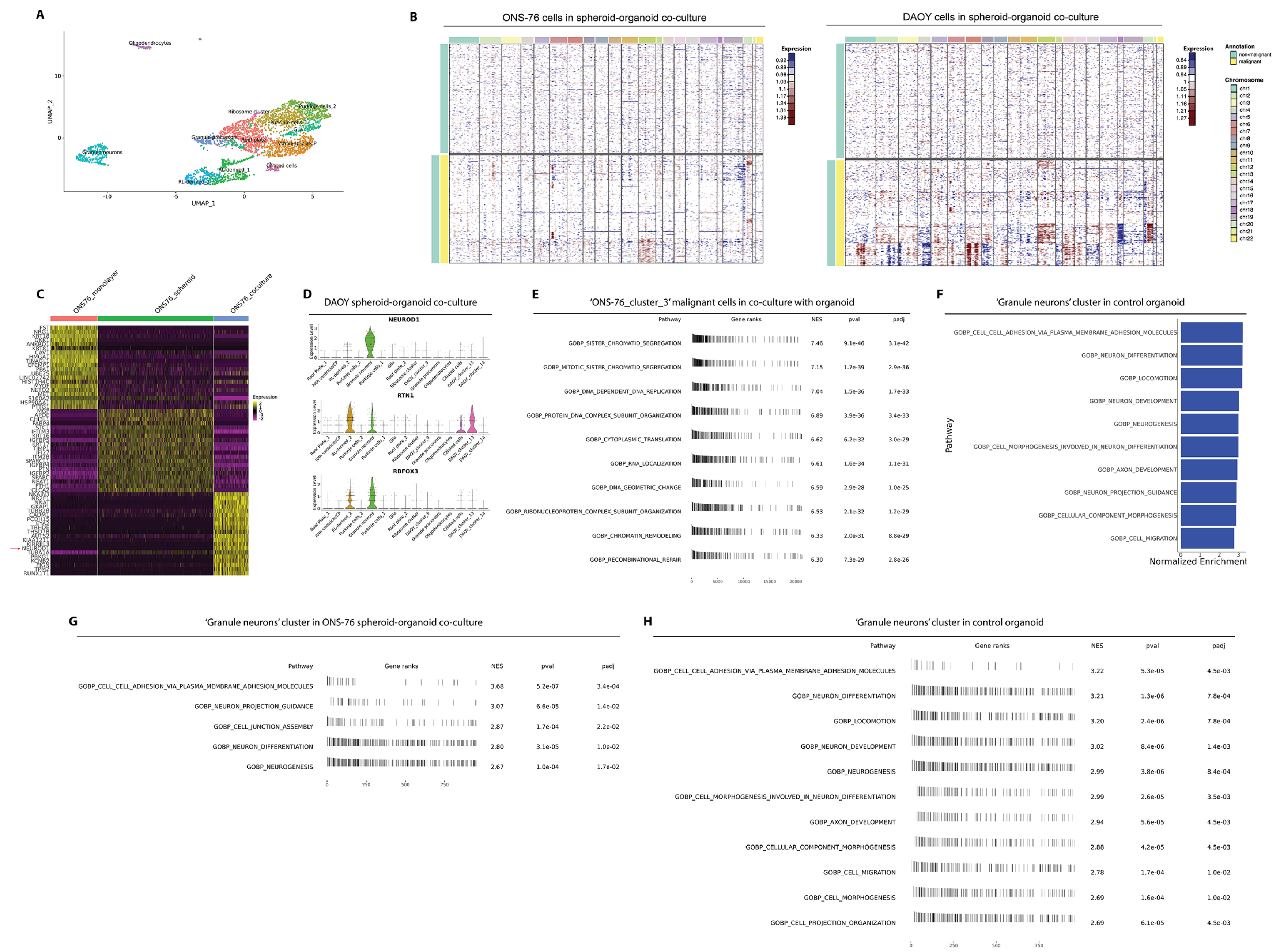
A
B
ONS-76 cells in spheroid-organoid co-culture
DAOY cells in spheroid-organoid co-culture
C
D
DAOY spheroid-organoid co-culture
E
'ONS-76_cluster_3' malignant cells in co-culture with organoid
F
'Granule neurons' cluster in control organoid
G
'Granule neurons' cluster in ONS-76 spheroid-organoid co-culture
H
'Granule neurons' cluster in control organoid

Supplementary Figure S3. Functional enrichment of GO biological pathways (GOBP) in ONS-76 malignant cells in tumour spheroid-organoid co-culture. (A) UMAP visualisation of single cell transcriptomes in non-malignant control organoid. (B) InferCNV analysis of copy number alterations in ONS-76 (left) and DAOY (right) spheroid-organoid co-cultures. (C) Heatmap of top twenty differentially expressed genes in ONS-76 monolayer, spheroid and malignant co-culture cells. Non-malignant cells in co-culture were not included. *NEUROD1* expression is indicated (red arrow). (D) Expression of *NEUROD1* in the DAOY tumour spheroid-organoid co-culture sample is restricted to the non-malignant 'Granule neurons' cluster. (E) GOBP enrichment scores and associated false discovery rate values (padj) in 'ONS76_cluster_3' cells in tumour spheroid-organoid co-culture. (F) fgsea geneset enrichment of upregulated GO biological pathway terms evaluated by normalised enrichment scores in the 'Granule neurons' cluster of the control organoid. (G) GOBP enrichment scores and associated padj values in the 'Granule neurons' cluster in tumour spheroid-organoid co-culture. (H) GOBP enrichment scores and associated padj values in the 'Granule neurons' cluster of the control organoid.

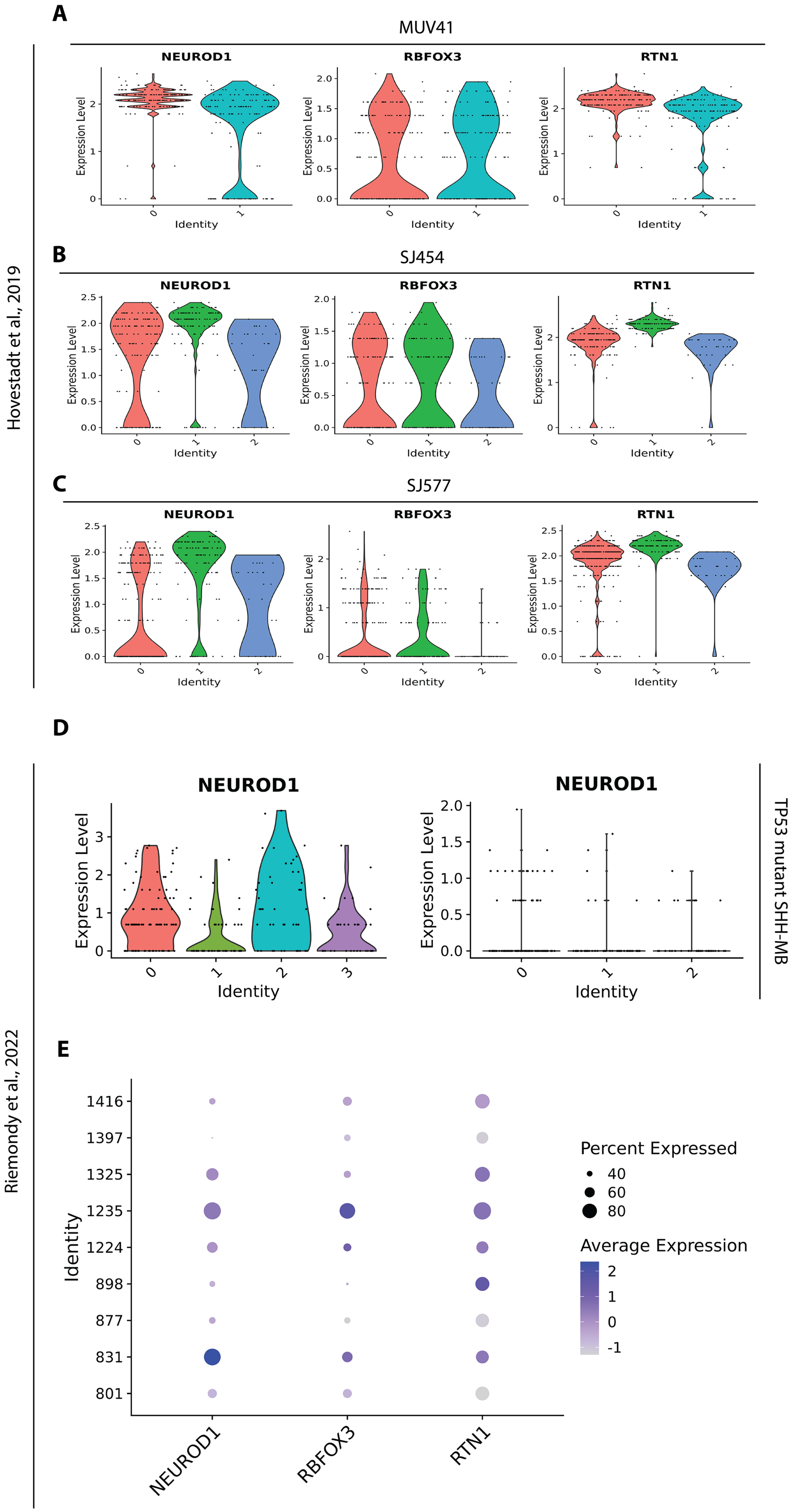
A
MUV41
NEUROD1
RBFOX3
RTN1
Expression Level
Identity
B
SJ454
NEUROD1
RBFOX3
RTN1
Expression Level
Identity
C
SJ577
NEUROD1
RBFOX3
RTN1
Expression Level
Identity
Hovestadt et al., 2019
D
NEUROD1
NEUROD1
Expression Level
Identity
TP53 mutant SHH-MB
E
Identity
1416
1397
1325
1235
1224
898
877
831
801
NEUROD1
RBFOX3
RTN1
Percent Expressed
40
60
80
Average Expression
2
1
0
-1
Riemondy et al., 2022

Supplementary Figure S4. Differentiating granule cell markers in SHH-MB tumours sequenced at single cell level across two public datasets.[1,2] (A) Violin plot of expression of the indicated markers in tumour sample MUV41 (n = 338 tumour cells).[1] (B) Violin plot of expression of the indicated markers in tumour sample SJ454 (n = 293 tumour cells).[1] (C) Violin plot of expression of the indicated markers in tumour sample SJ577 (n = 493 tumour cells).[1] (D) Violin plot of expression of *NEUROD1* in two *TP53* mutant tumours.[2] Left panel tumour ID: 801, right panel tumour ID: 1397. (E) Dot plot of the indicated granule cell differentiation markers expressed in nine SHH-medulloblastoma tumours (represented by identities 801 – 1416 on the y-axis).[2]

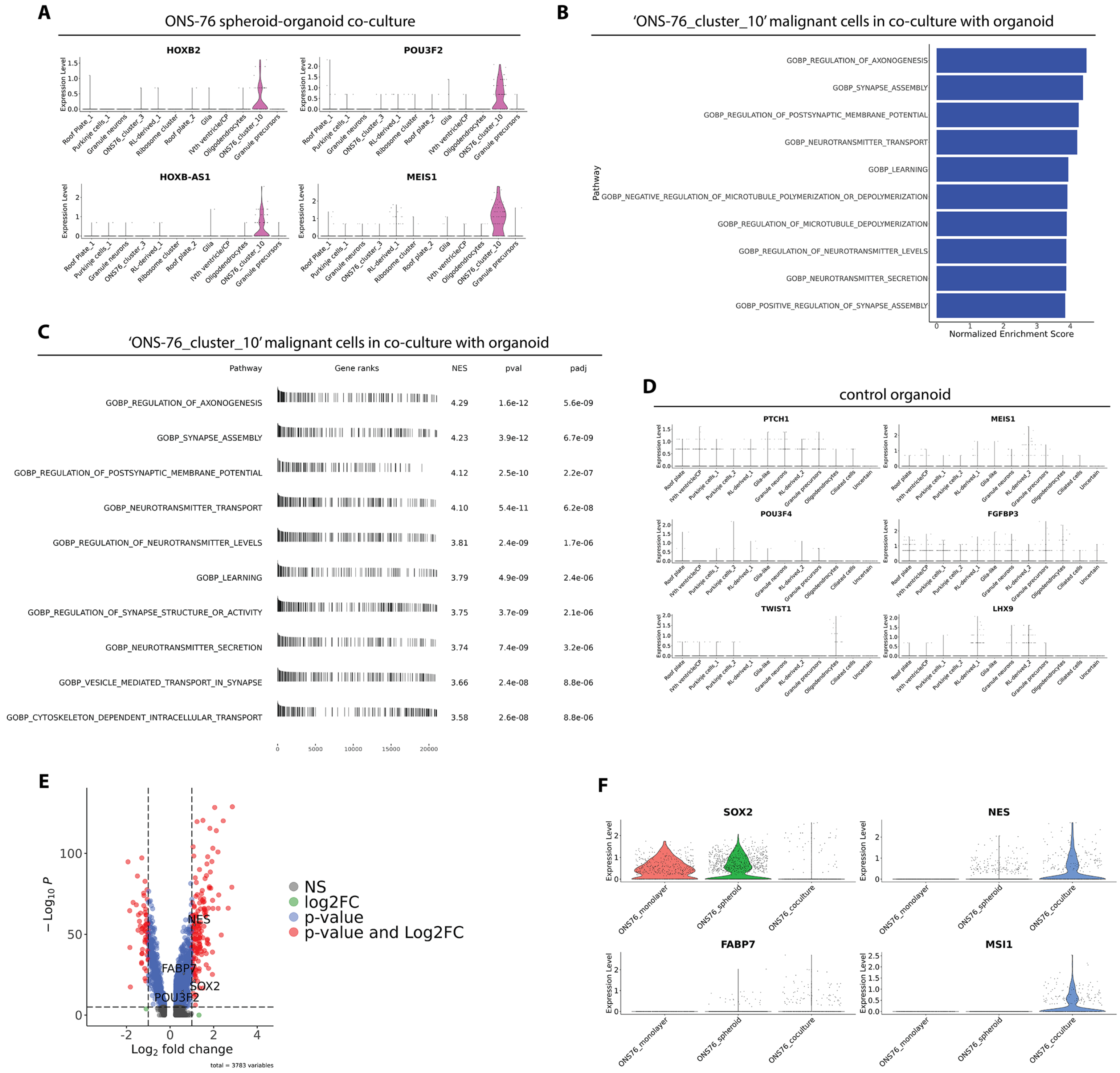
A
ONS-76 spheroid-organoid co-culture
HOXB2
POU3F2
HOXB-AS1
MEIS1
Expression Level
Roof Plate_1
Purkinje cells_1
Granule neurons
ONS76_cluster_3
RL-derived_1
Ribosome cluster
Roof plate_2
Glia
IVth ventricle/CP
Oligodendrocytes
ONS76_cluster_10
Granule precursors
B
'ONS-76_cluster_10' malignant cells in co-culture with organoid
Pathway
GOBP_REGULATION_OF_AXONOGENESIS
GOBP_SYNAPSE_ASSEMBLY
GOBP_REGULATION_OF_POSTSYNAPTIC_MEMBRANE_POTENTIAL
GOBP_NEUROTRANSMITTER_TRANSPORT
GOBP_LEARNING
GOBP_NEGATIVE_REGULATION_OF_MICROTUBULE_POLYMERIZATION_OR_DEPOLYMERIZATION
GOBP_REGULATION_OF_MICROTUBULE_DEPOLYMERIZATION
GOBP_REGULATION_OF_NEUROTRANSMITTER_LEVELS
GOBP_NEUROTRANSMITTER_SECRETION
GOBP_POSITIVE_REGULATION_OF_SYNAPSE_ASSEMBLY
Normalized Enrichment Score
C
'ONS-76_cluster_10' malignant cells in co-culture with organoid
Pathway | Gene ranks | NES | pval | padj
GOBP_REGULATION_OF_AXONOGENESIS | 4.29 | 1.6e-12 | 5.6e-09
GOBP_SYNAPSE_ASSEMBLY | 4.23 | 3.9e-12 | 6.7e-09
GOBP_REGULATION_OF_POSTSYNAPTIC_MEMBRANE_POTENTIAL | 4.12 | 2.5e-10 | 2.2e-07
GOBP_NEUROTRANSMITTER_TRANSPORT | 4.10 | 5.4e-11 | 6.2e-08
GOBP_REGULATION_OF_NEUROTRANSMITTER_LEVELS | 3.81 | 2.4e-09 | 1.7e-06
GOBP_LEARNING | 3.79 | 4.9e-09 | 2.4e-06
GOBP_REGULATION_OF_SYNAPSE_STRUCTURE_OR_ACTIVITY | 3.75 | 3.7e-09 | 2.1e-06
GOBP_NEUROTRANSMITTER_SECRETION | 3.74 | 7.4e-09 | 3.2e-06
GOBP_VESICLE_MEDIATED_TRANSPORT_IN_SYNAPSE | 3.66 | 2.4e-08 | 8.8e-06
GOBP_CYTOSKELETON_DEPENDENT_INTRACELLULAR_TRANSPORT | 3.58 | 2.6e-08 | 8.8e-06
0 5000 10000 15000 20000
D
control organoid
PTCH1
MEIS1
POU3F4
FGFBP3
TWIST1
LHX9
Roof plate
IVth ventricle/CP
Purkinje cells_1
Purkinje cells_2
RL-derived_1
Glia-like
Granule neurons
RL-derived_2
Granule precursors
Oligodendrocytes
Ciliated cells
Uncertain
E
$-\text{Log}_{10}\ P$
Log_2 fold change
NES
FABP7
SOX2
POU3F2
NS
log2FC
p-value
p-value and Log2FC
total = 3783 variables
F
SOX2
NES
FABP7
MSI1
Expression Level
ONS76_monolayer
ONS76_spheroid
ONS76_coculture

Supplementary Figure S5. Gene expression in ONS-76 and DAOY cells in spheroid-organoid co-culture. (A) Violin plot of marker genes of the second malignant cluster, 'ONS76_cluster_10' in the ONS-76 tumour spheroid-organoid co-culture sample. (B) fgsea geneset enrichment of upregulated GO biological pathway terms evaluated by normalised enrichment scores in 'ONS_cluster_10' of the tumour spheroid-organoid co-culture. (C) GOBP enrichment scores and associated padj values in 'ONS76_cluster_10' cells in tumour spheroid-organoid co-culture. (D) Expression of the indicated genes in cells of the control organoid sample. (E) Volcano plot of differentially expressed genes in DAOY cells in tumour spheroid-organoid co-culture compared to DAOY tumour-spheroids. NSC marker genes (labelled) are upregulated. (F) Violin plots of expression of the indicated neural stem cell markers in ONS-76 cells in monolayer culture, tumour spheroid culture and tumour spheroid-organoid co-culture.

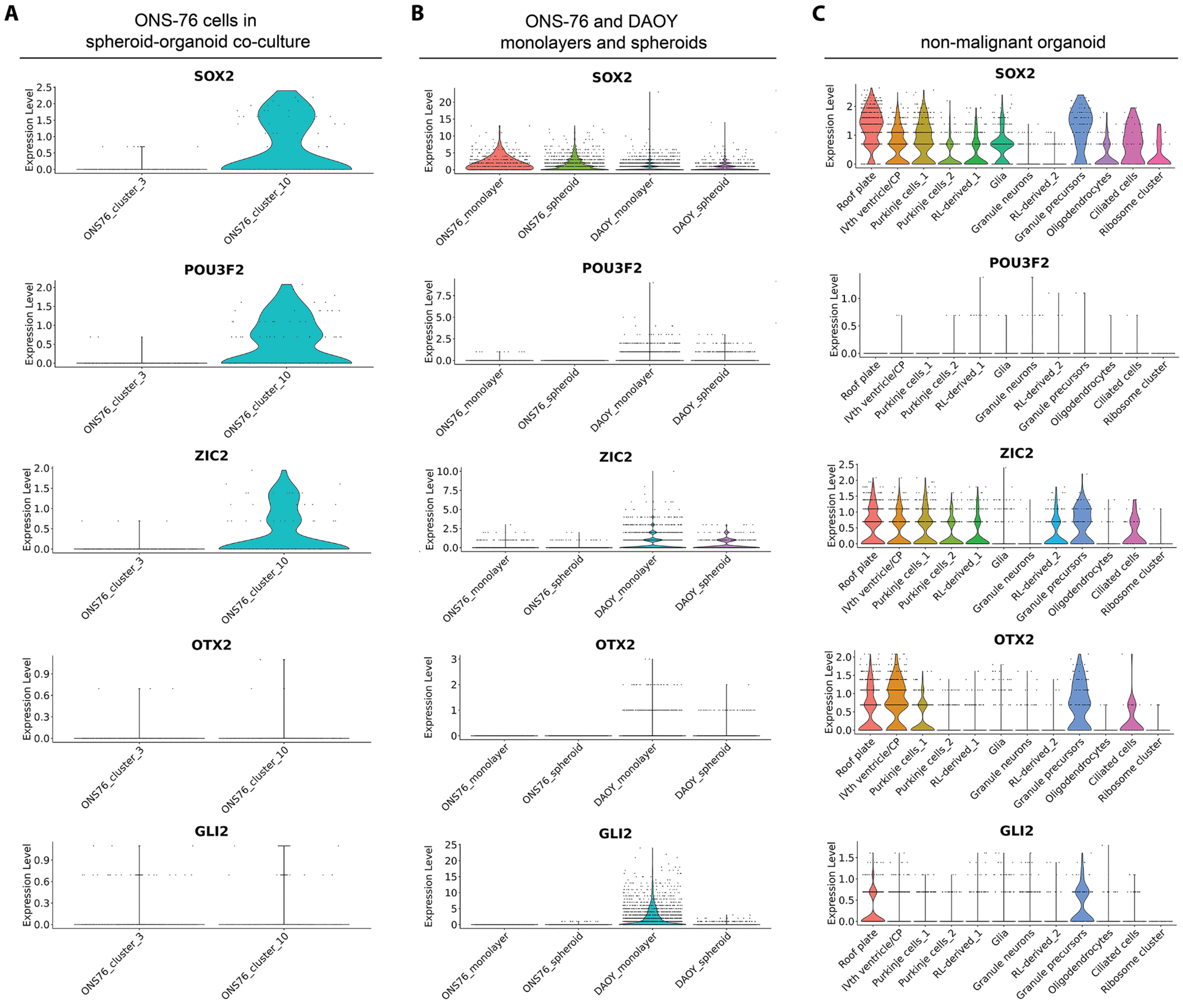
A
ONS-76 cells in
spheroid-organoid co-culture
B
ONS-76 and DAOY
monolayers and spheroids
C
non-malignant organoid
SOX2
POU3F2
ZIC2
OTX2
GLI2
Expression Level
ONS76_cluster_3
ONS76_cluster_10
ONS76_monolayer
ONS76_spheroid
DAOY_monolayer
DAOY_spheroid
Roof plate
IVth ventricle/CP
Purkinje cells_1
Purkinje cells_2
RL-derived_1
Glia
Granule neurons
RL-derived_2
Granule precursors
Oligodendrocytes
Ciliated cells
Ribosome cluster

Supplementary Figure S6. Expression of a sparse *SOX2* regulatory network of genes in control tumour samples. (A) Expression of the indicated genes in ONS-76 cells in co-culture. (B) *SOX2* regulatory network expression in ONS-76 and DAOY monolayer and tumour spheroid cultures. (C) Gene expression of the *SOX2* regulatory network in the non-malignant, control organoid.

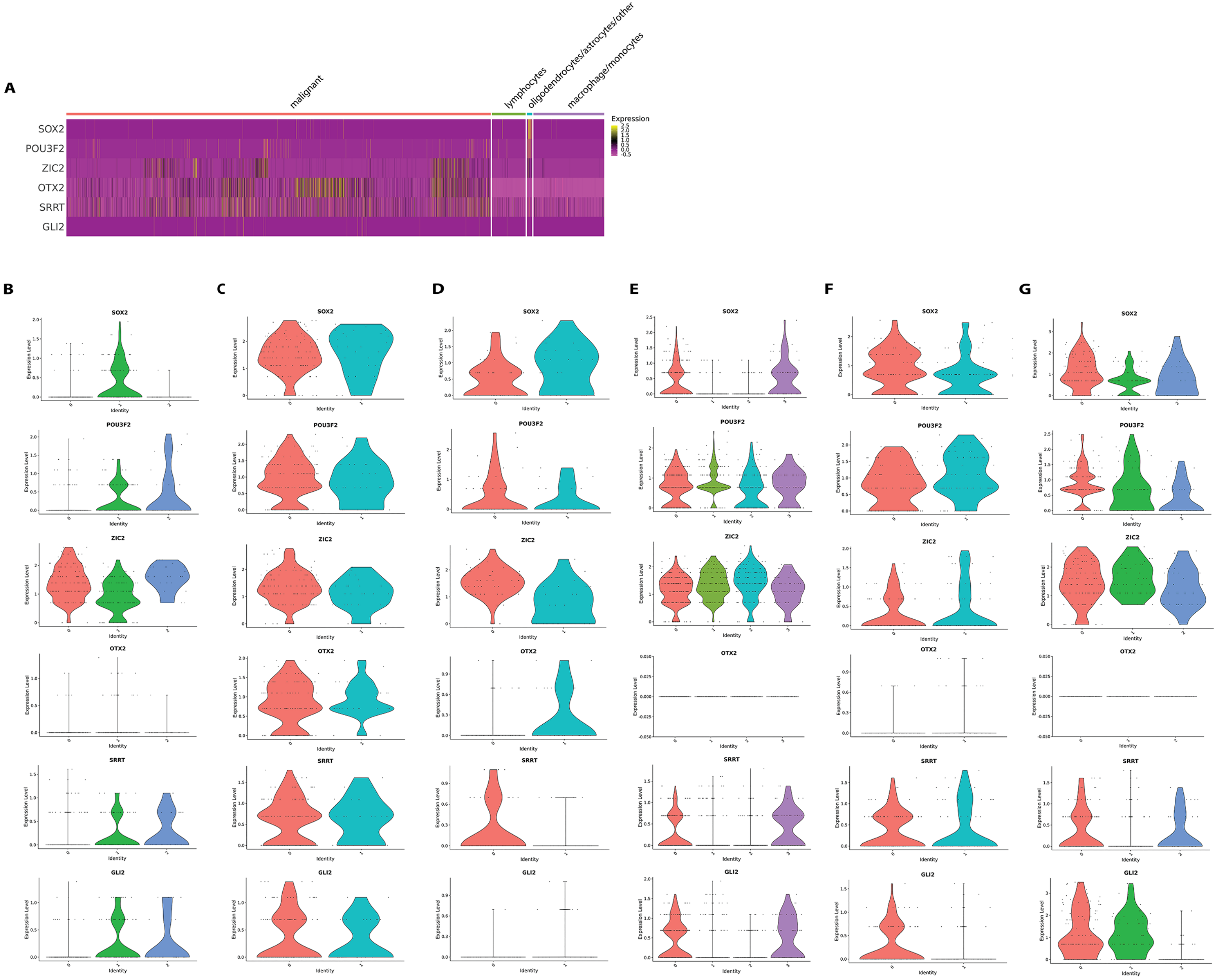
A
malignant
lymphocytes
oligodendrocytes/astrocytes/other
macrophage/monocytes
Expression
SOX2
POU3F2
ZIC2
OTX2
SRRT
GLI2
B
C
D
E
F
G
Expression Level
Identity

Supplementary Figure S7. Expression of a *SOX2* regulatory network in patient SHH-MB tumours but not in other MB subtypes. (A) Heatmap of the expression in single cells of the *SOX2* regulatory network for Group 3, Group 4 and WNT malignant and non-malignant cells. Cell labels are retained from the original publication. (B – G) Expression of the *SOX2* regulatory network in transcriptional cell clusters from a second dataset.[2] B, SHH-MB tumour ID: 831 (n = 264 tumour cells); C, SHH-MB tumour ID: 877 (n = 264 tumour cells); D, SHH-MB tumour ID: 898 (n = 66 tumour cells); E, SHH-MB tumour ID: 1325 (n = 538 tumour cells); F, SHH-MB tumour ID: 1416 (n = 144 tumour cells). G, SHH-MB tumour ID: 1397 (n = 198 tumour cells, *TP53* mutant).

References

1. Hovestadt V, Smith KS, Bihannic L, Filbin MG, Shaw ML, Baumgartner A, DeWitt JC, Groves A, Mayr L, Weisman HR, Richman AR, Shore ME, Goumnerova L, Rosencrance C, Carter RA, Phoenix TN, Hadley JL, Tong Y, Houston J, Ashmun RA, DeCuypere M, Sharma T, Flasch D, Silkov A, Ligon KL, Pomeroy SL, Rivera MN, Rozenblatt-Rosen O, Rusert JM, Wechsler-Reya RJ, Li X-N, Peyrl A, Gojo J, Kirchhofer D, Lötsch D, Czech T, Dorfer C, Haberler C, Geyeregger R, Halfmann A, Gawad C, Easton J, Pfister SM, Regev A, Gajjar A, Orr BA, Slavc I, Robinson GW, Bernstein BE, Suvà ML, Northcott PA. Resolving medulloblastoma cellular architecture by single-cell genomics. *Nature.* 2019; 572(7767):74-79.

2. Riemondy KA, Venkataraman S, Willard N, Nellan A, Sanford B, Griesinger AM, Amani V, Mitra S, Hankinson TC, Handler MH, Sill M, Ocasio J, Weir SJ, Malawsky DS, Gershon TR, Garancher A, Wechsler-Reya RJ, Hesselberth JR, Foreman NK, Donson AM, Vibhakar R. Neoplastic and immune single-cell transcriptomics define subgroup-specific intra-tumoral heterogeneity of childhood medulloblastoma. *Neuro Oncol.* 2022; 24(2):273-286.
