# Supplementary figures and images for: Sonic hedgehog medulloblastoma cells in co-culture with cerebellar organoids converge towards in vivo malignant cell states
Source: Neurooncol Adv. 2024 Dec 13;7(1):vdae218. doi: 10.1093/noajnl/vdae218 (PMC11783571; doi:10.1093/noajnl/vdae218)

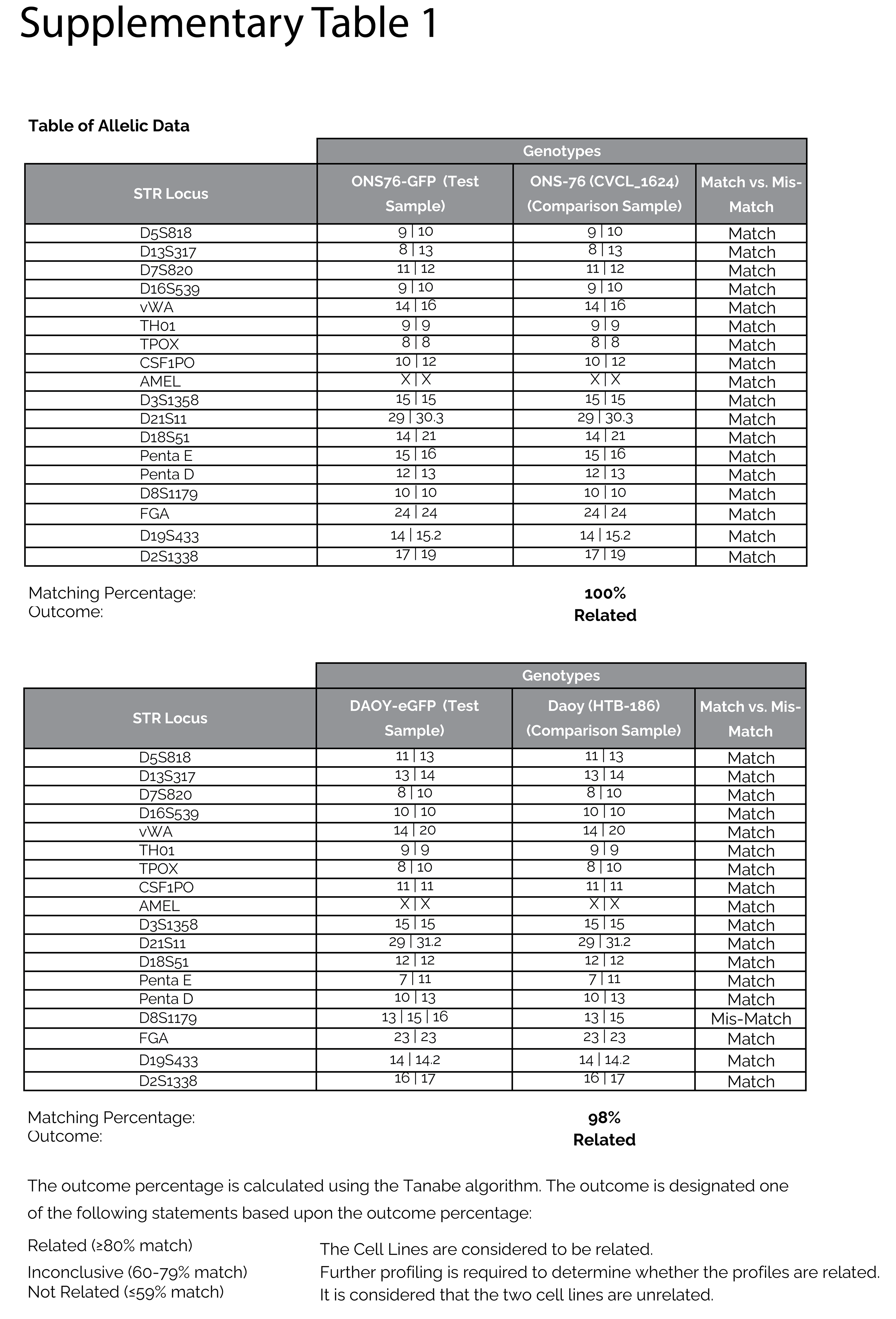

Supplement: vdae218_suppl_Supplementary_Tables_S1-S8_Figures_S1-S7 [file vdae218_suppl_supplementary_tables_s1-s8_figures_s1-s7.zip › Supplementary Table 1.tif]
